# Single Nucleotide Variants of Candidate Genes in Aggrecan Metabolic Pathway Are Associated with Lumbar Disc Degeneration and Modic Changes

**DOI:** 10.1371/journal.pone.0169835

**Published:** 2017-01-12

**Authors:** Romain Shanil Perera, Poruwalage Harsha Dissanayake, Upul Senarath, Lalith Sirimevan Wijayaratne, Aranjan Lional Karunanayake, Vajira Harshadeva Weerabaddana Dissanayake

**Affiliations:** 1 Department of Allied Health Sciences, Faculty of Medicine, University of Colombo, Colombo, Sri Lanka; 2 Department of Anatomy, Faculty of Medical Sciences, University of Sri Jayewardenepura, Gangodawila, Nugegoda, Sri Lanka; 3 Department of Community Medicine, Faculty of Medicine, University of Colombo, Colombo, Sri Lanka; 4 National Hospital of Sri Lanka, Colombo, Sri Lanka; 5 Department of Anatomy, Faculty of Medicine, University of Kelaniya, Ragama, Sri Lanka; 6 Human Genetics Unit, Department of Anatomy, Faculty of Medicine, University of Colombo, Colombo, Sri Lanka; University of Northampton, UNITED KINGDOM

## Abstract

**Introduction:**

Lumbar disc degeneration (LDD) is genetically determined and severity of LDD is associated with Modic changes. Aggrecan is a major proteoglycan in the intervertebral disc and end plate. Progressive reduction of aggrecan is a main feature of LDD and Modic changes.

**Objectives:**

The study investigated the associations of single nucleotide variants (SNVs) of candidate genes in the aggrecan metabolic pathway with the severity of LDD and Modic changes. In-silico functional analysis of significant SNVs was also assessed.

**Methods:**

A descriptive cross sectional study was carried out on 106 patients with chronic mechanical low back pain. T1, T2 sagittal lumbar MRI scans were used to assess the severity of LDD and Modic changes. 62 SNVs in ten candidate genes (*ACAN*, *IL1A*, *IL1B*, *IL6*, *MMP3*, *ADAMTS4*, *ADAMTS5*, *TIMP1*, *TIMP2* and *TIMP3*) were genotyped on Sequenom MassARRAY iPLEX platform. Multiple linear regression analysis was carried out using PLINK 1.9 in accordance with additive genetic model. In-silico functional analysis was carried out using Provean, SIFT, PolyPhen and Mutation Taster.

**Results:**

Mean age was 52.42±9.42 years. 74 (69.8%) were females. The rs2856836, rs1304037, rs17561 and rs1800587 variants of the *IL1A* gene were associated with the severity of LDD and Modic changes. The rs41270041 variant of the *ADAMTS4* gene and the rs226794 variant of the *ADAMTS5* gene were associated with severity of LDD while the rs34884997 variant of the *ADAMTS4* gene, the rs55933916 variant of the *ADAMTS5* gene and the rs9862 variant of the *TIMP3* gene were associated with severity of Modic changes. The rs17561 variant of the *IL1A* gene was predicted as pathogenic by the PolyPhen prediction tool.

**Conclusions:**

SNVs of candidate genes in *ACAN* metabolic pathway are associated with severity of LDD and Modic changes in patients with chronic mechanical low back pain. Predictions of in-silico functional analysis of significant SNVs are inconsistent.

## Introduction

Chronic low back pain is the leading cause of years lived with disability in most regions of the world including South Asia [1]. Most of the chronic low back pain is due to mechanical causes such as strains, sprains and complications related to lumbar disc degeneration (LDD) [2]. Lumbar intervertebral disc consists of central nucleus pulposus, surrounding annulus fibrosus and cartilage end plate. LDD is characterised by the structural failure and cell mediated response to the changes in the composition of the disc [3]. Modic changes are types of degenerative changes which involve the vertebral end plate and bone marrow [4] and are also associated with the severity of LDD and chronic low back pain [5]. There is a wide variation in the severity of LDD/Modic changes in the population as some individuals develop early and severe types of LDD/Modic changes compared to other individuals in the same age category. With the recent advancement in genetic research, it has been suggested that these degenerative changes are genetically determined and are modified to some degree by behavioural and environmental factors [6, 7].

Genetics can determine the size, shape and mechanical properties of the spinal structures including the intervertebral disc. LDD is characterised by structural damage to the disc matrix and increased activity of matrix degrading enzymes [8]. In addition, persistent inflammation and subsequent vascular and neural responses are key features of painful LDD and Modic changes [9, 10]. Therefore the structural components and molecules in their degradation pathways are ideal candidates for genetic association studies. Aggrecan (coded by the *ACAN* gene) is a large molecular weight proteoglycan with numerous glycosaminoglycan side chains and is the core proteoglycan in the disc matrix and vertebral end plate. A Variable Number of Tandem Repeat (VNTR) polymorphism in exon 12 of the *ACAN* gene is associated with LDD where individuals with shorter number of tandem repeats are at higher risk for severe LDD [11–14]. There are a few studies which have assessed the associations of single nucleotide variants (SNVs) of the *ACAN* gene with the severity of LDD, however most of them are intronic variants [15, 16].

Candidate genes involved in metabolic pathways of LDD and Modic changes (eg. interleukins, matrix metalloproteinases, aggrecanases and tissue inhibitors of metalloproteinases) are also implicated with the onset and degree of mechanical failure of the intervertebral disc and vertebral end plate. Interleukins (IL1α, IL1β and IL6) are a group of local cytokines involved in the regulation of the inflammatory response. The “T” allele of the rs1800587 variant of the *IL1A* gene (codes the IL1α molecule) is associated with early LDD in girls [17] and Modic changes [18]. Matrix metalloproteinase 3 (MMP3 and is coded by the *MMP3* gene) is the most frequently studied metalloproteinase and the 5A allele of the rs3025058 variant of the *MMP3* gene is associated with progression and severity of LDD [19–21]. A disintegrin and metalloproteinase with thrombospondin motifs 4 and 5 (ADAMTS 4 and ADAMTS 5 and are coded by the *ADAMTS4* and *ADAMTS5* genes, respectively) are the main aggrecanases involved in degrading the protein core of aggrecan [22]. The “T” allele of the rs4233367 variant of the *ADAMTS4* has a lower risk for LDD [23]. In addition, the “A” allele of the rs151058 variant, “A” allele of the rs229052 variant and “A” allele of the rs162502 variant of the *ADAMTS5* gene (intronic variants) are associated with LDD [24]. Tissue inhibitors of metalloproteinases (TIMPs) are a group of protease inhibitors which inhibit the catabolic actions of MMPs and ADAMTSs [25] and the C124T variant of the *TIMP1* gene is associated with radiographic progression of LDD [20].

Intensity of pain and severity of disability of the chronic mechanical low back pain are correlated with severity of LDD [26] and Modic changes [5]. Severity of LDD and Modic changes might be affected by the SNVs of the molecules involved in the metabolic pathway of the *ACAN* gene. However most of the identified SNVs associated with LDD/Modic changes are located at the intronic regions of the DNA sequence and evidence for SNVs in exonic regions of the respective candidate genes is limited. ADAMTSs and TIMPs are relatively recently found molecules and there are very few studies which have investigated the associations of SNVs of genes of these molecules with the severity of LDD/Modic changes [25]. Although the associations of SNVs of candidate genes are identified, evidence of their functional involvement in LDD/Modic changes are not well established. The main objective of this study was to determine the associations of single nucleotide variants of candidate genes in the aggrecan metabolic pathway, with the severity of LDD and Modic changes in patients with chronic mechanical low back pain. We also assessed the in-silico functional analysis of the significant SNVs associated with the severity of LDD/Modic changes.

## Methods

### Study design, setting and participants

A descriptive cross-sectional study was conducted on patients with chronic mechanical low back pain who attended the rheumatology clinic, National Hospital of Sri Lanka, Colombo. Both male and female patients of Sri Lankan origin with chronic mechanical low back pain aged 20 to 69 years were included in the study based on the severity of disc related degenerative changes in the x-rays of lumbar spine. Low back pain was defined as pain, muscle tension, or stiffness localized below the costal margin and above the inferior gluteal folds, with or without radiating pain to the leg [27]. Low back pain during day time worsening in the latter part of the day due to movements such as bending, lifting, walking, running, standing and sitting were considered as being due to a mechanical cause [28, 29]. Chronicity was defined as pain on most days of the week of at least three months duration [30]. Patients with back pain due to inflammatory causes, visceral origin, systemic infections affecting spine, metabolic bone diseases, fractures in the vertebral column, past surgeries in the spine, and spinal tumours were excluded. Pregnant females and patients who refused to participate in the study were also excluded.

**Fig 1:**
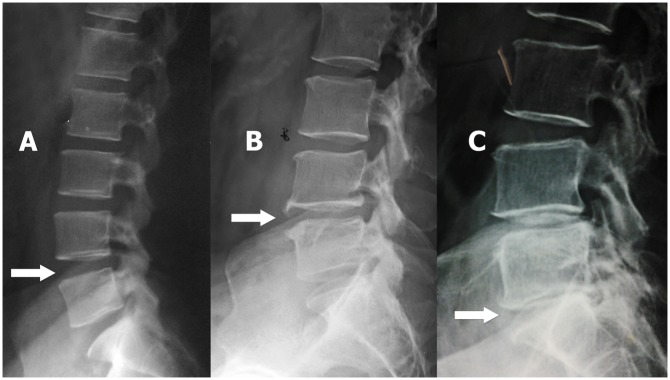
Assessment of the x-ray features of lumbar disc degeneration—lateral x-ray of lumbar spine. Arrows—A—no disc space narrowing / anterior osteophytes (grade 0 LDD); B—mild disc space narrowing and small anterior osteophytes (grade 1 LDD); C—small anterior osteophytes and moderate disc space narrowing (grade 2 LDD).

Three hundred and sixty eight patients with chronic mechanical low back pain who attended the rheumatology clinic, National Hospital of Sri Lanka, Colombo from May 2012 to October 2014 underwent lateral x-ray of lumbar spine and x-rays were evaluated by a consultant radiologist blinded to the clinical details of the patients. Disc space narrowing and anterior osteophytes (L1/L2 to L5/S1) were graded and overall LDD was derived using a scoring system defined by Lane *et al*. 1993 (Fig 1). Each lumbar level was graded from grade 0 to grade 2 LDD [31]. Hundred and twenty patients were selected to undergo MRI scan of the lumbar spine based on following criteria. Patients with grade 2 LDD on lateral x-ray of lumbar spine were further assessed using mid-sagittal MRI scans of the lumbar spine. In addition, age and gender matched patients from other two groups of LDD (grade 0 LDD and grade 1 LDD) were also assessed using MRI scans of the lumbar spine for comparison purposes. The study was carried out in accordance with the Declaration of Helsinki and with the approval of the Ethics Review Committee of the Faculty of Medicine, University of Colombo. Written informed consent was obtained from the eligible patients before recruitment.

### Clinical evaluation

Age, gender and body mass index (BMI) were recorded using a pretested interviewer administered questionnaire and clinical examination. Height (cm) and weight (kg) of the patients were recorded with light clothing and without shoes to the nearest 0.1 cm and 0.1 kg, respectively, and BMI was calculated (kg/m^2^) [32]. International cut off values were used for categorisation of BMI [33].

### Radiological assessment

**Fig 2:**
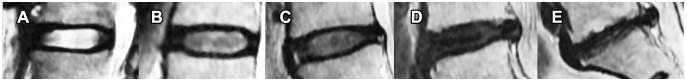
Grading system for assessment of MRI features of lumbar disc degeneration. Grade 1 (A)—homogeneous disc structure, normal disc height and distinction of nucleus and annulus is clear; grade 2 (B)—nonhomogeneous shape with horizontal bands, some blurring between nucleus and annulus; grade 3 (C)—nonhomogeneous shape with blurring between nucleus and annulus, shape of the annulus still recognizable; grade 4 (D)—nonhomogeneous shape with hypointensity, distinction between nucleus and annulus impossible, disc height usually decreased; grade 5 (E)—same as grade 4 but with a collapsed disc space.

T1 and T2 weighted sagittal MRI scans of lumbar spine were performed in supine position using a GE 1.5T MRI Scanner (Signa, General Electric, Milwaukee, Wisconsin). Patients underwent fast spin-echo sagittal T1 and T2 weighted imaging with slice thickness of 4 mm and interslice gap of 1 mm. MRI scans were assessed for the presence and severity of LDD and Modic changes from L1/L2 to L5/S1 levels by a consultant radiologist blinded to the clinical details of the patients. The severity of LDD was assessed on T2-weighted MRI images using the modified Pfirrmann grading system (Fig 2) and were graded as grade 1 (normal disc shape, no horizontal bands, distinction of nucleus and annulus is clear), grade 2 (nonhomogeneous shape with horizontal bands, some blurring between nucleus and annulus), grade 3 (nonhomogeneous shape with blurring between nucleus and annulus, shape of the annulus still recognizable), grade 4 (nonhomogeneous shape with hypointensity, distinction between nucleus and annulus impossible, disc height usually decreased), and grade 5 (same as grade 4 but with a collapsed disc space) [34, 35]. A particular grade of LDD was identified for each of the lumbar levels, and scores of five lumbar levels were summed to calculate the severity of LDD.

**Fig 3:**
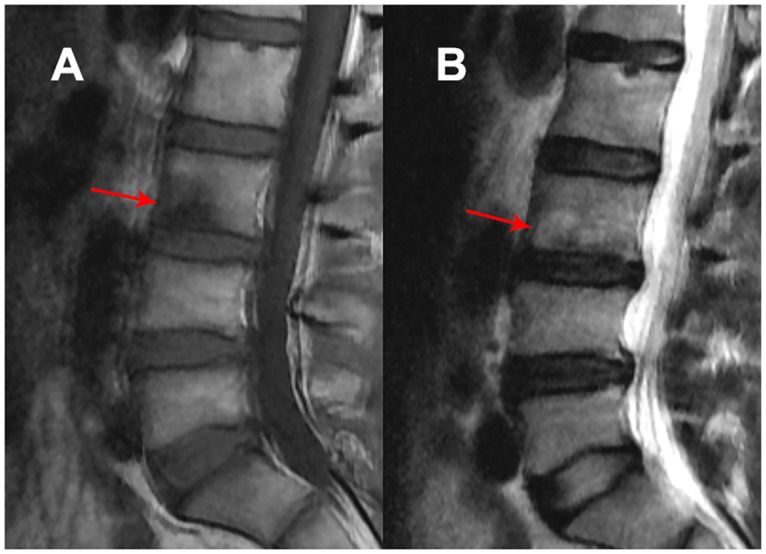
Type I Modic changes in lumbar spine. Hypointense changes in T1-weighted images (A) and hyperintense changes in T2 weighted MRI images (B).

**Fig 4:**
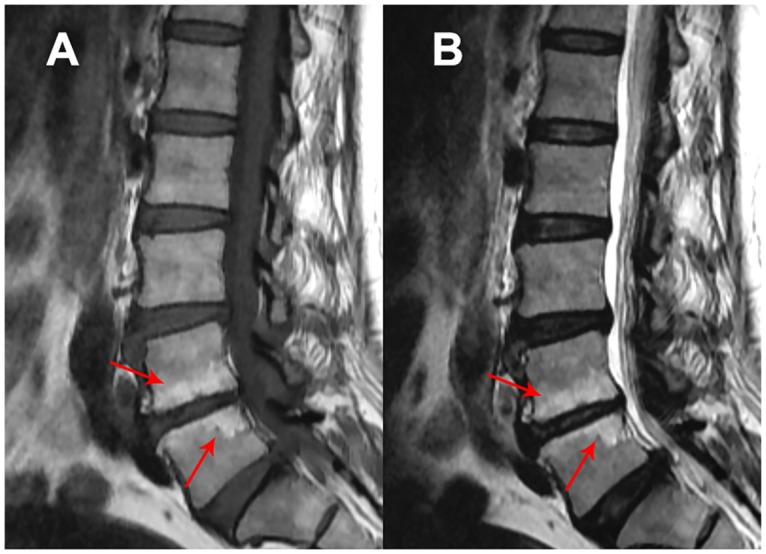
Type II Modic changes in lumbar spine. Hyperintense changes in both T1 (A) and T2 (B) weighted images.

**Fig 5:**
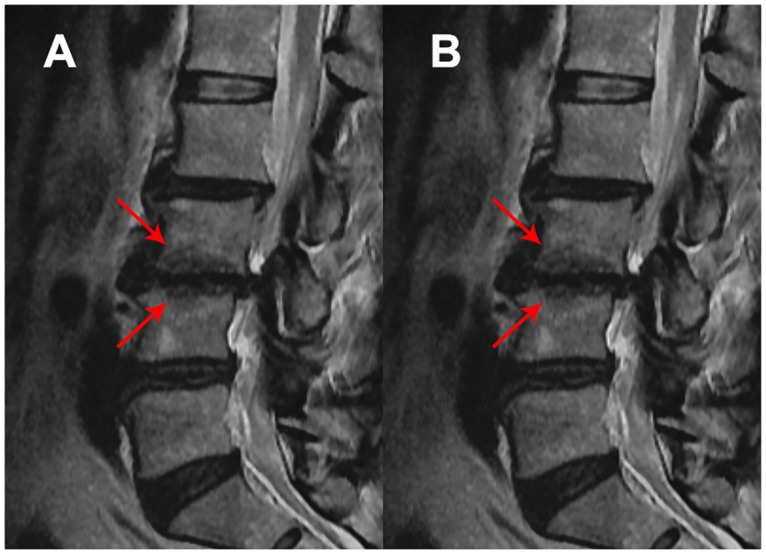
Type III Modic changes in lumbar spine. Hypointense changes in T1 (A) and T2 (B) weighted images.

Modic changes were recorded using both T1 and T2 weighted MRI images where vertebral end plate and bone marrow changes were scored as absent (no modic changes), type 1 (hypointense changes in T1-weighted images and hyperintense changes in T2 weighted images), type II (hyperintense changes in T1 and T2 weighted images) and type III (hypointense changes in T1 and T2 weighted images) (Figs 3–5) [4, 36]. Presence of type I, type II and type III Modic changes were given a score of 1, 2 and 3, respectively. The scores of each lumbar level were summed to get the severity of Modic changes.

### Selection of single nucleotide variants of candidate genes

The *ACAN* gene and candidate genes involved in the metabolic pathway of aggrecan were selected for the study (*IL1A*, *IL1B*, *IL6*, *MMP3*, *ADAMTS4*, *ADAMTS5*, *TIMP1*, *TIMP2* and *TIMP3*). Aggrecan and molecules involved in aggrecan metabolic pathway are summarised with their functions and respective gene symbols in Table 1. The SNVs of these genes are involved in regulation of aggrecan metabolism and other regulatory functions of the extracellular matrix (ECM). Common SNVs (minor allele frequency ≥ 0.05) in the exonic and untranslated regions (UTRs) of selected genes in twenty five Sri Lankan exomes were identified using an in-house variant calling pipeline at the Human Genetics Unit, Faculty of Medicine, University of Colombo. In addition, common variants in exonic and UTRs of the selected candidate genes among Gujarati Indians in Houston (GIH) were extracted from the HapMap database [37] (HapMap database provides human genetic variations among several global ancestry groups and GIH ancestry is the genetically most similar ancestry to the Sri Lankan population). Sixty two SNVs in exonic regions and UTRs from 10 genes were selected for genotyping (Table 2). Although there is a VNTR polymorphism and other intronic variants which are associated with the severity of LDD and Modic changes, this study was confined to exonic and UTR SNVs of the candidate genes.

**Table 1:** Selected genes which are involved in aggrecan and its metabolic pathway.

Component	Molecule	Gene symbol	Function
**Structural**	Aggrecan	*ACAN*	Main proteoglycan of the extracellular matrix and provides osmotic properties with the help of glycosaminoglycan side chains which absorbs and retains water
**Inflammatory**	Interleukin 1α	*IL1A*	Reduces the synthesis of collagen type I, II and aggrecan
	Interleukin 1β	*IL1B*	Inhibits nucleus pulposus cell proliferation and increases the synthesis of catabolic enzymes of aggrecan
	Interleukin 6	*IL6*	Protective role in non degenerated cells—controls the actions of IL1-β and TNF-α; Inflammatory role in degenerated cells—increases the synthesis of IL1-β and TNF-α
**Catabolic**	Matrix metalloproteinases 3	*MMP3*	Digests protein core of the aggrecan and small non collagenous proteins
	A disintegrin and metalloproteinase with thrombospondin motifs 4	*ADAMTS4*	Major aggrecanase which is involved in degradation of aggrecan core protein
	A disintegrin and metalloproteinase with thrombospondin motifs 5	*ADAMTS5*	Major aggrecanase which is involved in degradation of aggrecan core protein
**Anti catabolic**	Tissue inhibitors of metalloproteinase 1	*TIMP1*	Inhibits the catabolic actions of MMPs and ADAMTS
	Tissue inhibitors of metalloproteinase 2	*TIMP2*	Inhibits the catabolic actions of MMPs and ADAMTS
	Tissue inhibitors of metalloproteinase 3	*TIMP3*	Inhibits the catabolic actions of MMPs and ADAMTS

**Table 2:** Allele frequencies and Hardy–Weinberg equilibrium of genetic variants of candidate genes in aggrecan metabolic pathway.

No.	CHR	Position GRCh37/hg19	Gene	Region/function	SNV	Minor allele	Major allele	MAF (%)	HWE (p value)
1	1	161160872	*ADAMTS4*	3’ UTR	rs34884997	C	T	0.12	0.68
2	1	161161284	*ADAMTS4*	nonsynonymous	rs41270041	C	G	0.18	0.52
3	1	161163037	*ADAMTS4*	nonsynonymous	rs4233367	T	C	0.18	0.76
4	1	161168004	*ADAMTS4*	synonymous	rs33941127	T	C	0.40	0.85
5	1	161168189	*ADAMTS4*	nonsynonymous	rs34448954	T	C	0.13	0.42
6	2	113532083	*IL1A*	3’ UTR	rs2856836	G	A	0.27	0.06
7	2	113532236	*IL1A*	3’ UTR	rs1304037	C	T	0.28	0.06
8	2	113537223	*IL1A*	nonsynonymous	rs17561	A	C	0.27	0.06
9	2	113542960	*IL1A*	5’ UTR	rs1800587	A	G	0.27	0.06
10	2	113587121	*IL1B*	downstream	rs2853550	A	G	0.29	0.65
11	2	113590390	*IL1B*	synonymous	rs1143634	A	G	0.13	0.69
12	7	22766246	*IL6*	upstream	rs1800796	C	G	0.46	0.59
13	7	22766645	*IL6*	upstream	rs1800795	C	G	0.13	0.10
14	7	22771156	*IL6*	synonymous	rs2069849	T	C	0.05	1.00
15	11	102709425	*MMP3*	synonymous	rs520540	A	G	0.33	0.10
16	11	102713465	*MMP3*	synonymous	rs602128	A	G	0.33	0.10
17	11	102713620	*MMP3*	nonsynonymous	rs679620	T	C	0.33	0.21
18	15	89382027	*ACAN*	synonymous	rs372041880	C	A	0.07	0.10
19	15	89382129	*ACAN*	nonsynonymous	rs16942318	A	C	0.05	0.05
20	15	89386652	*ACAN*	nonsynonymous	rs34949187	A	G	0.11	1.00
21	15	89388894	*ACAN*	nonsynonymous	rs148070768	G	A	0.14	0.26
22	15	89388905	*ACAN*	synonymous	rs16942341	T	C	0.07	1.00
23	15	89391160	*ACAN*	synonymous	rs2272023	C	A	0.34	0.42
24	15	89392689	*ACAN*	nonsynonymous	rs144501729	A	C	0.14	0.26
25	15	89398105	*ACAN*	synonymous	rs2351491	T	C	0.37	0.84
26	15	89398553	*ACAN*	nonsynonymous	rs35430524	A	C	0.05	0.05
27	15	89398553	*ACAN*	nonsynonymous	rs3743399	G	A	0.28	0.65
28	15	89398631	*ACAN*	nonsynonymous	rs938609	A	T	0.37	0.70
29	15	89400339	*ACAN*	nonsynonymous	rs2882676	A	C	0.47	0.46
30	15	89400680	*ACAN*	nonsynonymous	rs28407189	G	A	0.07	1.00
31	15	89400963	*ACAN*	nonsynonymous	rs79925540	T	G	0.16	0.48
32	15	89401109	*ACAN*	nonsynonymous	rs4932439	A	G	0.28	0.65
33	15	89401615	*ACAN*	synonymous	rs3825994	G	T	0.44	0.85
34	15	89401616	*ACAN*	nonsynonymous	rs76282091	C	G	0.16	0.48
35	15	89402051	*ACAN*	nonsynonymous	rs1042630	A	G	0.41	0.45
36	15	89402239	*ACAN*	synonymous	rs1042631	T	C	0.35	1.00
37	15	89402596	*ACAN*	synonymous	rs698621	G	T	0.38	0.85
38	15	89415247	*ACAN*	nonsynonymous	rs3817428	G	C	0.05	1.00
39	15	89417238	*ACAN*	nonsynonymous	rs1126823	G	A	0.36	0.84
40	17	76867017	*TIMP2*	synonymous	rs2277698	T	C	0.24	0.80
41	21	28291455	*ADAMTS5*	3’ UTR	rs1444269	G	A	0.26	1.00
42	21	28291846	*ADAMTS5*	3’ UTR	rs2298657	C	T	0.05	1.00
43	21	28292581	*ADAMTS5*	3’ UTR	rs3746836	A	G	0.25	0.81
44	21	28293095	*ADAMTS5*	3’ UTR	rs229072	T	A	0.48	0.27
45	21	28293117	*ADAMTS5*	3’ UTR	rs229073	G	A	0.48	0.27
46	21	28293924	*ADAMTS5*	3’ UTR	rs11700721	T	C	0.12	1.00
47	21	28294090	*ADAMTS5*	3’ UTR	rs16979423	G	T	0.14	0.70
48	21	28294143	*ADAMTS5*	3’ UTR	rs9978597	G	T	0.04	1.00
49	21	28296135	*ADAMTS5*	3’ UTR	rs229078	T	G	0.22	1.00
50	21	28296324	*ADAMTS5*	3’ UTR	rs151065	A	G	0.19	0.56
51	21	28296389	*ADAMTS5*	synonymous	rs3746839	G	A	0.08	1.00
52	21	28302355	*ADAMTS5*	nonsynonymous	rs226794	A	G	0.10	0.60
53	21	28338298	*ADAMTS5*	nonsynonymous	rs457947	G	C	0.07	0.46
54	21	28338423	*ADAMTS5*	synonymous	rs55933916	G	C	0.08	0.50
55	22	33253280	*TIMP3*	synonymous	rs9862	T	C	0.50	0.46
56	22	33253292	*TIMP3*	synonymous	rs11547635	T	C	0.07	0.42
57	22	33257322	*TIMP3*	3’ UTR	rs1427384	C	T	0.19	0.53
58	22	33258050	*TIMP3*	3’ UTR	rs2267184	T	C	0.16	0.48
59	22	33258288	*TIMP3*	3’ UTR	rs1065314	C	T	0.17	0.32
60	23	47444879	*TIMP1*	nonsynonymous	rs5953060	C	G	0.43	0.66
61	23	47444985	*TIMP1*	synonymous	rs4898	C	T	0.43	0.66
62	23	47445286	*TIMP1*	nonsynonymous	rs6609533	G	A	0.43	0.66

CHR—chromosome number, SNV—single nucleotide variant, MAF—minor allele frequency, HWE—Hardy Weinberg equilibrium, UTR—untranslated region

### Sample collection, DNA extraction and quantification

Venous blood was drawn from a superficial forearm vein into an EDTA tube and stored at -20°C at the Human Genetics Unit, Faculty of Medicine, University of Colombo until processing. DNA was extracted from 200ml of venous blood using QIAamp DNA Mini Kit according to the blood and body fluid protocol (spin protocol) [38] summarised in S1 Protocol. Extracted DNA was quantified using the Quantus fluorometer (Promega) with QuantiFluor^®^ Double stranded DNA (dsDNA) system [39] summarised in S1 Protocol. Quantified DNA was diluted in water and normalised and optimized for the subsequent Sequenom MassARRAY system.

### Genotyping

All selected genetic variants were genotyped using Sequenom iPLEX MassARRAY system (Sequenom, San Diego, CA) [40] at the Australian Genome Research Facility, Australia. The workflow of the Sequenom iPLEX reaction included locus specific polymerase chain reaction, locus specific primer extension reaction and annealing of oligonucleotide primer at the upstream of the polymorphic site being genotyped. Then the primer and amplified target DNA were incubated with mass modified dideoxynucleotide terminators. The primer extension was made according to the sequence of the variant site. Mass of the extended primer was determined through the use of matrix-assisted laser desorption ionization time of flight mass spectrometry. The alleles present at the polymorphic site of interest were assessed based on the sequence specified by the mass of the primer. The mass of the observed primers were translated into a genotype by the SpectroTYPER software of the Sequenom system [40].

### Statistical analysis

Descriptive statistics were calculated to summarise the sample characteristics. Statistical analysis was carried out using PLINK 1.9 software [41]. Variants with Hardy–Weinberg equilibrium ≥ 0.05 were included into the analysis. Multiple linear regression analysis was used for assessing associations of quantitative outcomes (severity of LDD and severity of Modic changes) with SNVs of interest. The genotype of the respective SNV was treated as a quantitative variable and coded 0, 1 or 2 to represent the number of variant allele, consistent with an additive genetic model. Age, gender and BMI were used as the additional covariates. Permutation tests with 10,000 permutations were carried out to generate the significance levels empirically to relax the assumptions of normality and correct the errors due to small sample size. P value < 0.05 was used as the level of significance. In-silico functional analysis was carried out using four free online bioinformatics prediction tools: Provean [42], SIFT [43], PolyPhen [44] and Mutation taster [45].

## Results

Among hundred and twenty patients selected, only 106 patients attended for the MRI scanning. Sample characteristics of the 106 patients who underwent MRI scan of lumbar spine are summarised in Table 3. The majority of patients belonged to 50–59 years age category. Most of the patients were females. Twenty three of the females (31.1%) were obese while 4 males were obese (12.5%). There was moderate agreement between the x-ray scoring system and MRI scoring system of LDD (Cohen's kappa = 0.49). All 106 patients who underwent MRI assessment had atleast grade 2 LDD. Mean severity of LDD was 12.56 ± 2.88. Eighteen patients (17%) had Modic changes and type 2 Modic changes were common. Mean severity of Modic changes was 0.49 ± 1.22. Genotyping efficiency was more than 98% and minor allele frequency ranged from 0.04 to 0.5. All variants were in Hardy–Weinberg equilibrium (Table 2).

**Table 3:** Summary of the sample characteristics of 106 patients who underwent genetic association analysis.

Variable		N (%)
Total patients		106
Age	Mean	52.42 ± 9.42 yrs
	20–29 yrs	3 (2.8)
	30–39 yrs	7 (6.6)
	40–49 yrs	22 (20.8)
	50–59 yrs	51 (48.1)
	60–69 yrs	23 (21.7)
Gender	Female	74 (69.8)
	Male	32 (30.2)
BMI	Normal (18–24.9 kg/m^2^)	37 (34.9)
	Overweight (25–29.9 kg/m^2^)	42 (39.6)
	Obese (≥ 30 kg/m^2^)	27 (25.5)
LDD (maximum score)	Grade 1	0 (0.0)
	Grade 2	7 (6.6)
	Grade 3	56 (52.8)
	Grade 4	36 (34.0)
	Grade 5	7 (6.6)
Modic changes	Yes	18 (17.0)
	No	88 (83.0)
Types of Modic changes	I	1 (0.9)
	II	15 (14.2)
	III	2 (1.9)

LDD—lumbar disc degeneration, LDD (maximum score)–maximum grade of LDD in L1/L2 to L5/S1 levels of the respective spine, BMI—body mass index

### Associations of genetic variants with the severity of lumbar disc degeneration

*IL1A*, *ADAMTS4* and *ADAMTS5* genes were significantly associated with the severity of LDD (Table 4). Each additional “G” allele of the rs2856836 variant, “C” allele of the rs1304037 variant, “A” allele of the rs17561 variant and “A” allele of the rs1800587 variant of the *IL1A* gene were associated with progressive reduction in the severity of LDD. These four SNVs were in strong linkage disequilibrium with each other (R^2^ ≥ 0.94, D’ ≥ 0.98). The presence of each additional “C” allele of the rs41270041 variant of the *ADAMTS4* gene was associated with progressive reduction in severity of LDD. The presence of the “A” allele of the rs226794 variant of the *ADAMTS5* gene increased the severity of LDD.

**Table 4:** Severity of MRI features tabulated according to genotype and the results of multiple linear regression (significant SNVs)^a^.

No.	Gene	SNV	Variable	A1	MAF	A2/A2	A1/A2	A1/A1	Regression coefficient (β)	Adjusted p value
1	*ADAMTS4*	rs34884997		C	0.12					
			Genotypes			T/T	C/T	C/C		
			N			80	23	2		
			LDD mean (SD)			12.34 (2.97)	13.13 (2.56)	14.00 (2.83)	0.12	0.14
			Modic mean (SD)			0.30 (1.05)	1.04 (1.58)	2.00 (0.00)	0.31	0.00*
2	*ADAMTS4*	rs41270041		C	0.18					
			Genotypes			G/G	C/G	C/C		
			N			69	35	1		
			LDD mean (SD)			12.72 (2.73)	12.34 (3.10)	7.00 (0.00)	-0.22	0.01*
			Modic mean (SD)			0.51 (1.21)	0.49 (1.29)	0.00 (0.00)	-0.03	0.80
3	*IL1A*	rs2856836		G	0.27					
			Genotypes			A/A	G/A	G/G		
			N			58	38	9		
			LDD mean (SD)			13.09 (2.93)	11.92 (2.72)	11.67 (2.83)	-0.21	0.01*
			Modic mean (SD)			0.69 (1.46)	0.32 (0.87)	0.00 (0.00)	-0.20	0.04*
4	*IL1A*	rs1304037		C	0.28					
			Genotypes			T/T	C/T	C/C		
			N			58	37	10		
			LDD mean (SD)			13.09 (2.93)	11.97 (2.73)	11.50 (2.72)	-0.22	0.01*
			Modic mean (SD)			0.69 (1.46)	0.32 (0.88)	0.00 (0.00)	-0.20	0.04*
5	*IL1A*	rs17561		A	0.27					
			Genotypes			C/C	A/C	A/A		
			N			58	38	9		
			LDD mean (SD)			13.09 (2.93)	11.92 (2.72)	11.67 (2.83)	-0.21	0.01*
			Modic mean (SD)			0.69 (1.46)	0.32 (0.87)	0.00 (0.00)	-0.20	0.04*
6	*IL1A*	rs1800587		A	0.27					
			Genotypes			G/G	A/G	A/A		
			N			59	38	9		
			LDD mean (SD)			13.10 (2.91)	12.11 (2.82)	10.89 (2.03)	-0.23	0.00*
			Modic mean (SD)			0.68 (1.46)	0.32 (0.87)	0.00 (0.00)	-0.20	0.04*
7	*ADAMTS5*	rs226794		A	0.1					
			Genotypes			G/G	A/G	A/A		
			N			84	21	0		
			LDD mean (SD)			12.14 (2.92)	14.14 (2.15)	NA	0.20	0.02*
			Modic mean (SD)			0.48 (1.26)	0.57 (1.12)	NA	0.03	0.75
8	*ADAMTS5*	rs55933916		G	0.08					
			Genotypes			C/C	G/C	G/G		
			N			90	13	1		
			LDD mean (SD)			12.56 (2.92)	12.31 (3.87)	15.00 (0.00)	-0.02	0.82
			Modic mean (SD)			0.40 (1.08)	0.62 (1.26)	6.00 (0.00)	0.27	0.01*
9	*TIMP3*	rs9862		T	0.5					
			Genotypes			C/C	T/C	T/T		
			N			23	58	23		
			LDD mean (SD)			13.26 (2.42)	12.64 (3.06)	11.61 (2.78)	-0.15	0.07
			Modic mean (SD)			1.04 (1.58)	0.40 (1.15)	0.22 (0.85)	-0.23	0.02*

SNV—single nucleotide variant, LDD—lumbar disc degeneration, SD—standard deviation, A1 –minor allele, A2 –major allele, MAF—minor allele frequency, NA—not applicable, β–standardised regression coefficient, data were analysed by multiple linear regression on variant genotypes adjusting for age, gender and body mass index.

*—p value < 0.05

^a^ The tabulation of MRI features according to genotype of 62 SNVs and results of their multiple linear regression are summarised in S1 Table.

### Associations of genetic variants with the severity of Modic changes

*IL1A*, *ADAMTS4*, *ADAMTS5* and *TIMP3* genes were associated with the severity of Modic changes (Table 4). Each additional “G” allele of the rs2856836 variant, “C” allele of the rs1304037 variant, “A” allele of the rs17561 variant and “A” allele of the rs1800587 variant of the *IL1A* gene were associated with progressive reduction in severity of Modic changes. All four SNVs were in strong linkage disequilibrium as mentioned previously. The presence of each additional “C” allele of the rs34884997 variant of the *ADAMTS4* gene was associated with progressive increase in the severity of Modic changes. Each additional “G” allele of the rs55933916 variant of the *ADAMTS5* gene increased the severity of Modic changes. Furthermore, the presence of each additional “T” allele of the rs9862 variant of the *TIMP3* gene was associated with progressive reduction in the severity of Modic changes.

### In-silico functional analysis of the significant variants associated with the severity of lumbar disc degeneration and Modic changes

Among twenty four nonsynonymous variants, only three were associated with severity of LDD/Modic changes. Amino acid substitutions of the rs41270041 variant of the *ADAMTS4* gene and the rs226794 variant of the *ADAMTS5* gene are non conservative substitutions. Among the nine significant variants, only the rs17561 variant of the *IL1A* gene is predicted as pathogenic by the PolyPhen functional prediction tool. The rs55933916 variant of the *ADAMTS5* gene and the rs9862 variant of the *TIMP3* gene are located in conserved regions of the respective genes. However both these variants were predicted as non pathogenic by the four prediction tools (Table 5).

**Table 5:** Significant SNVs associated with severity of lumbar disc degeneration/Modic changes and their in-silico functional analysis.

No	Gene	SNV	Region/function	Significant associations	Amino acid change	Conservative substitution	Conserved region	Provean	SIFT	PolyPhen	Mutant Taster
1	*ADAMTS4*	rs34884997	3' UTR	Modic changes			No				Harmless
2		rs41270041	nonsynonymous	LDD	P720A	No	No	Neutral	Tolerated	Benign	Harmless
3	*IL1A*	rs2856836	3' UTR	LDD, Modic changes			No				Harmless
4		rs1304037	3' UTR	LDD, Modic changes			No				Harmless
5		rs17561	nonsynonymous	LDD, Modic changes	A114S	Yes	No	Neutral	Tolerated	**Damaging**	Harmless
6		rs1800587	5' UTR	LDD, Modic changes							
7	*ADAMTS5*	rs226794	nonsynonymous	LDD	L692P	No	No	Neutral	Tolerated	Benign	Harmless
8		rs55933916	synonymous	Modic changes			Yes	Neutral	Tolerated	Benign	Harmless
9	*TIMP3*	rs9862	synonymous	Modic changes			Yes	Neutral	Tolerated	Benign	Harmless

SNV—single nucleotide variant, LDD—lumbar intervertebral disc degeneration, UTR—untranslated regions, Mutant taster (polymorphism automatic) = Harmless, AA—amino acid

## Discussion

In this study we assessed the associations of single nucleotide variants of the candidate genes in the metabolic pathway of the aggrecan gene with the severity of LDD and Modic changes. In addition we performed the in-silico functional analysis of the variants significantly associated with the severity of LDD and Modic changes. We found that, the “G” allele of the rs2856836 variant, the “C” allele of the rs1304037 variant, the “A” allele of the rs17561 variant and the “A” allele of the rs1800587 variant of the *IL1A* gene to be associated with the severity of LDD and Modic changes. In addition the “C” allele of the rs41270041 variant of the *ADAMTS4* gene and the “A” allele of the rs226794 variant of the *ADAMTS5* gene were associated with the severity of LDD. The “C” allele of the rs34884997 variant of the *ADAMTS4* gene, the “G” allele of the rs55933916 variant of the *ADAMTS5* gene and the “T” allele of the rs9862 variant of the *TIMP3* gene were associated with the severity of Modic changes. The rs17561 variant of the *IL1A* gene was predicted as pathogenic by the PolyPhen functional prediction tool.

LDD is a complex phenomenon and it was previously considered that LDD was totally age dependent. With the advancement of genetic research, genetic associations have become the main determining factor for initiation and progression of LDD [46]. Intervertebral discs with unfavourable genetic variations are weak and matrix can be easily disrupted due to activities of normal daily living [47]. Furthermore, functions of metabolic pathways (inflammatory, catabolic and anti-catabolic pathways) of structural components of the intervertebral disc can be affected by SNVs of candidate genes [7, 48]. With LDD, normal balance of the biomechanical forces of the spine are disrupted and reflected on the nearby structures including the vertebrae and ligaments [3]. Modic changes are a type of degenerative changes which involve the end plate and bone marrow of the vertebrae and are well correlated with the severity of LDD [36]. In our sample, severity of Modic changes correlated positively with the severity of LDD (correlation coefficient (r) = 0.29, p < 0.01).

Aggrecan is a large aggregating proteoglycan and a main structural component of the intervertebral disc and vertebral end plate [49]. Any factor that alter the synthesis and function of the aggrecan in the extracellular matrix would make the disc vulnerable to biomechanical forces leading to increased secretion of catabolic enzymes and subsequent degeneration [50]. In our study, variants of the *ACAN* gene were not associated with the severity of LDD or Modic changes based on the additive genetic model. Most published studies have identified an association of VNTR polymorphism in exon 12 of the *ACAN* gene with LDD [11–13]. However, the studies which have focused on associations of SNVs of exonic regions of the *ACAN* gene with LDD are limited [15, 16]. The “T” allele of the rs1042631 variant of the *ACAN* gene was associated with reduced signal intensity in a population based cohort of 588 Finnish males [16]. The Finnish study graded the LDD based on intensity of the disc signal, but the modified Pfirrmann grading system which we used includes additional variables to grade LDD such as homogeneity of the disc, distinction between nucleus and annulus and height of disc [36]. Therefore discrepancies in scoring systems may have influenced the results.

It has been postulated that persistent inflammation and subsequent vascular and nerve ingrowths are the key factors associated with painful LDD [9] and Modic changes [6]. SNVs of the *IL1A* gene may modify the function of the IL1α resulting altered synthesis of ECM molecules including aggrecan. According to published literature, the “T’ allele of the rs1800587 variant of the *IL1A* gene is associated with Modic changes in middle aged men among Finnish occupational groups (machine drivers, carpenters and office workers) [18]. It is also associated with the early onset of LDD in Danish young girls (12–14 yrs) [51]. The “C” and “T” alleles are the recorded allele combination in the Finnish population, but our sample had “G” and “A” allele combination. However, the genotype frequencies are compatible with the GIH ancestry in HapMap database. According to our results, the presence of each additional “A” allele of the rs1800587 variant reduced the severity of LDD and Modic changes. The rs2856836, rs1304037, rs17561 and rs1800587 variants of the *IL1A* gene are in strong linkage disequilibrium and share similar minor allele frequency and functional properties. Similarly, the presence of each additional minor allele of the rs2856836, rs1304037 and rs17561 variants reduced the severity of LDD and Modic changes. These variants have been identified as associated factors in inflammatory arthritis. Specifically, the rs2856836 variant and the rs17561 variant of the *IL1A* gene are associated with ankylosing spondylitis [52]. The rs17561 variant and the rs1800587 variant of *IL1A* gene are associated with erosive/aggressive arthritis and the rs1304037 variant of the *IL1A* gene is associated with rheumatoid arthritis [53]. However there are no previous reports on the association between degenerative features of the spine and rs2856836, rs1304037, and rs17561 variants of the *IL1A* gene.

The rs2856836, rs1304037 and rs1800587 variants were located at UTRs of the *IL1A* gene and were predicted as non pathogenic by the mutation taster tool. The rs17561 variant is a non-synonymous variant located in exon 5 of the *IL1A* gene and causes an Alanine to Serine substitution at position 114 of the amino acid sequence. This substitution is a highly conservative substitution and is located at a non conserved region of the *IL1A* gene. Highly conservative substitutions are not considered to be pathogenic. Likewise variants located at non conserved regions of the genes are also less likely to be pathogenic. In our study, one prediction tool predicted the rs17561 variant as pathogenic (Provean—neutral; SIFT—tolerated; PolyPhen—possibly damaging; Mutation taster: polymorphism automatic). However, it involves in splice site regulation and alters the features of the final protein (IL1α) according to the Mutation Taster. As overall results are inconsistent, it is important to confirm its functional role with functional genetic studies.

ADAMTS4 and ADAMTS5 are main aggrecanases which degrade the protein core of the aggrecan molecule leading to matrix breakdown of the intervertebral disc and vertebral end plate [22, 54]. According to animal studies increased expression of the *ADAMTS4* gene accelerates the extracellular matrix breakdown and reduces synthesis of proteoglycan in the intervertebral disc [55, 56]. Each additional “C” allele of the rs41270041 variant of the *ADAMTS4* gene reduced the severity of LDD in our sample, but this SNV did not have a significant association with LDD in the Chinese Han Population [23]. The rs41270041 variant is a non-synonymous variant located in exon 9 of the *ADAMTS4* gene and causes a Proline to Alanine substitution at position 720 of the amino acid sequence, but it does not affect the overall function of the *ADAMTS4* gene according to in-silico functional analysis. The rs34884997 variant is a 3’ UTR variant of the *ADAMTS4* gene and the “C” allele was associated with increased severity of Modic changes, but there was no evidence with regard to this SNV in the literature.

The evidence for association of SNVs of exonic regions of the *ADAMTS5* gene with LDD/Modic changes is limited [24]. The presence of the “A” allele of the rs226794 variant of the *ADAMTS5* gene increased the severity of LDD in our patients. This SNV has been studied for its association with Achilles tendon pathology and knee osteoarthritis, but none of the studies had strong evidence to support the hypothesis [57, 58]. The rs226794 variant is a non-synonymous variant located in exon 7 of the *ADAMTS5* gene and causes a Leucine to Proline substitution at position 692 of the amino acid sequence. Although it causes a non conservative substitution, in-silico functional analysis was unremarkable. In addition, the presence of each additional “G” allele of the rs55933916 variant of the *ADAMTS5* gene increased the severity of Modic changes. Minor allele frequency of the “G” allele of the rs55933916 variant of the *ADAMTS5* gene is 0.08, but there was only one patient with the “GG” genotype in our sample and this patient had a comparatively higher mean severity in LDD and Modic changes. The rs55933916 variant is a synonymous variant and there were no records on this SNV in the GIH ancestry in the HapMap database. Although it does not change the amino acid sequence, it is involved in splice site variation and causes subsequent changes in the protein features. However, the rs226794 and rs55933916 variants do not affect the overall function of the ADAMTS5 protein according to in-silico functional analysis. There were no previous records on both of these variants of the *ADAMTS5* gene with regard to its association with LDD/Modic change.

TIMPs are regulatory proteins which inhibit the local actions of MMP3, ADAMTS4 and ADAMTS5 molecules during the process of LDD. Although there are three TIMP molecules which are involved in LDD/Modic changes, only the “T” allele of the rs9862 variant of the *TIMP3* gene was associated with a progressive reduction in the severity of Modic changes in our sample. Male oral cancer patients in Taiwan who carried the “T” allele of the rs9862 variant of the *TIMP3* gene had increased plasma levels of TIMP3 molecule [59]. Therefore the “T” allele of the rs9862 variant of the *TIMP3* gene may have increased the production of TIMP3 molecules resulting in increased inhibition of ADAMTS4 and ADAMTS5 molecules causing subsequent reduction of aggrecan degradation. However the rs9862 variant is a synonymous variant and results of the in-silico analysis were unremarkable.

Quantitative traits like severity of LDD and Modic changes may have cumulative/additive effects from multiple variants from several candidate genes. In addition there may be a significant effect from the environmental factors on the expression of the respective genetic variants [7]. We have not corrected the genetic association analysis for the multiple variants we already assessed and other probable variants in other respective genes. Only one of the significant variants predicted as pathogenic by the PolyPhen prediction tool and results of the other prediction tools were inconsistent. Therefore studies using a larger sample number with whole genome sequencing may be required to identify the significant SNVs associated with these quantitative traits and will need to be followed by functional genetic studies. However this study provides an initial basis for identifying probable SNVs which are associated with the severity of LDD and Modic changes.

### Limitations of the study

This study was conducted on a specific group of patients with chronic mechanical low back pain and the results are not generalisable. Although the study was conducted at one centre, it represented patients from three districts of the Western Province of Sri Lanka. Low sample number is another limitation and the study had a 80% power to detect significant p values of regression coefficients ≥ 0.25 according to “Quanto” software programme [60]. However, with the use of permutation function in PLINK software tool and other covariates (age, gender, and BMI), the study detected significant p values of regression coefficients ≥ 0.2. There may be other confounding factors for LDD and Modic changes such as occupation, type of physical activity which we have not corrected for.

### Conclusions

Variants of the *IL1A*, *ADAMTS4* and ADAMTS5 genes are associated with the severity of LDD and Modic changes. In addition, a variant of the *TIMP3* gene is associated with the severity of Modic changes. The degenerative findings of the spine are not a mere product of ageing process. Although nine SNVs have significant associations with LDD and Modic changes, only the rs17561 variant of the *IL1A* gene is predicted as pathogenic by in-silico functional analysis. Generally many of the other SNVs are acting as genetic markers for probable functional variants in the locations elsewhere in the same gene or nearby genes.

## Supporting Information

S1 Protocol DNA extraction and quantification methodology. (DOCX)

S1 Table The tabulation of MRI features according to genotype of 62 SNVs and results of their multiple linear regression. (DOCX)

S1 Data Genotype data of 106 patients. (XLSX)

## References

[1] Vos T, Flaxman AD, Naghavi M, Lozano R, Michaud C, Ezzati M, et al Years lived with disability (YLDs) for 1160 sequelae of 289 diseases and injuries 1990–2010: a systematic analysis for the Global Burden of Disease Study 2010. Lancet. 2012;380(9859):2163–2196. 10.1016/S0140-6736(12)61729-2 23245607 PMC6350784

[2] Deyo RA, Weinstein JN. Low back pain. N Engl J Med. 2001;344(5):363–370. 10.1056/NEJM200102013440508 11172169

[3] Modic MT, Ross JS. Lumbar degenerative disk disease. Radiology. 2007;245(1):43–61. 10.1148/radiol.2451051706 17885180

[4] Modic MT, Steinberg PM, Ross JS, Masaryk TJ, Carter JR. Degenerative disk disease: assessment of changes in vertebral body marrow with MR imaging. Radiology. 1988;166(1 Pt 1):193–199. 10.1148/radiology.166.1.3336678 3336678

[5] Mok FP, Samartzis D, Karppinen J, Fong DY, Luk KD, Cheung KM. Modic changes of the lumbar spine: prevalence, risk factors, and association with disc degeneration and low back pain in a large-scale population-based cohort. The spine journal: official journal of the North American Spine Society. 2016;16(1):32–41. 26456851 10.1016/j.spinee.2015.09.060

[6] Karppinen J, Daavittila I, Solovieva S, Kuisma M, Taimela S, Natri A, et al Genetic factors are associated with modic changes in endplates of lumbar vertebral bodies. Spine. 2008;33(11):1236–1241. 10.1097/BRS.0b013e318170fd0e 18469698

[7] Kalichman L, Hunter DJ. The genetics of intervertebral disc degeneration. Associated genes. Joint Bone Spine. 2008;75(4):388–396. 10.1016/j.jbspin.2007.11.002 18485784

[8] Adams MA, Dolan P. Intervertebral disc degeneration: evidence for two distinct phenotypes. J Anat. 2012;221(6):497–506. 10.1111/j.1469-7580.2012.01551.x 22881295 PMC3512277

[9] Peng Y, Lv FJ. Symptomatic versus Asymptomatic Intervertebral Disc Degeneration: Is Inflammation the Key? Critical reviews in eukaryotic gene expression. 2015;25(1):13–21. 25955814 10.1615/critreveukaryotgeneexpr.2015012369

[10] Lotz JC, Fields AJ, Liebenberg EC. The role of the vertebral end plate in low back pain. Global spine journal. 2013;3(3):153–164. 10.1055/s-0033-1347298 24436866 PMC3854605

[11] Kim NK, Shin DA, Han IB, Yoo EH, Kim SH, Chung SS. The association of aggrecan gene polymorphism with the risk of intervertebral disc degeneration. Acta Neurochir (Wien). 2011;153(1):129–133. 20936487 10.1007/s00701-010-0831-2

[12] Kawaguchi Y, Osada R, Kanamori M, Ishihara H, Ohmori K, Matsui H, et al Association between an aggrecan gene polymorphism and lumbar disc degeneration. Spine. 1999;24(23):2456–2460. 10626307 10.1097/00007632-199912010-00006

[13] Gu J, Guan F, Guan G, Xu G, Wang X, Zhao W, et al Aggrecan variable number of tandem repeat polymorphism and lumbar disc degeneration: a meta-analysis. Spine. 2013;38(25):E1600–1607. 10.1097/BRS.0000000000000012 24296484

[14] Cong L, Pang H, Xuan D, Tu GJ. Association between the expression of aggrecan and the distribution of aggrecan gene variable number of tandem repeats with symptomatic lumbar disc herniation in Chinese Han of Northern China. Spine. 2010;35(14):1371–1376. 10.1097/BRS.0b013e3181c4e022 20505571

[15] Rajasekaran S, Kanna RM, Senthil N, Raveendran M, Cheung KM, Chan D, et al Phenotype variations affect genetic association studies of degenerative disc disease: conclusions of analysis of genetic association of 58 single nucleotide polymorphisms with highly specific phenotypes for disc degeneration in 332 subjects. The spine journal: official journal of the North American Spine Society. 2013;13(10):1309–1320. 23792102 10.1016/j.spinee.2013.05.019

[16] Videman T, Saarela J, Kaprio J, Nakki A, Levalahti E, Gill K, et al Associations of 25 structural, degradative, and inflammatory candidate genes with lumbar disc desiccation, bulging, and height narrowing. Arthritis Rheum. 2009;60(2):470–481. 10.1002/art.24268 19180518

[17] Eskola PJ, Kjaer P, Daavittila IM, Solovieva S, Okuloff A, Sorensen JS, et al Genetic risk factors of disc degeneration among 12-14-year-old Danish children: a population study. International journal of molecular epidemiology and genetics. 2010;1(2):158–165. 21537388 PMC3076758

[18] Karppinen J, Solovieva S, Luoma K, Raininko R, Leino-Arjas P, Riihimaki H. Modic changes and interleukin 1 gene locus polymorphisms in occupational cohort of middle-aged men. Eur Spine J. 2009;18(12):1963–1970. 10.1007/s00586-009-1139-x 19701653 PMC2899448

[19] Yuan HY, Tang Y, Liang YX, Lei L, Xiao GB, Wang S, et al Matrix metalloproteinase-3 and vitamin d receptor genetic polymorphisms, and their interactions with occupational exposure in lumbar disc degeneration. Journal of occupational health. 2010;52(1):23–30. 20009418 10.1539/joh.l8149

[20] Valdes AM, Hassett G, Hart DJ, Spector TD. Radiographic progression of lumbar spine disc degeneration is influenced by variation at inflammatory genes: a candidate SNP association study in the Chingford cohort. Spine. 2005;30(21):2445–2451. 16261124 10.1097/01.brs.0000184369.79744.a5

[21] Omair A, Holden M, Lie BA, Reikeras O, Brox JI. Treatment outcome of chronic low back pain and radiographic lumbar disc degeneration are associated with inflammatory and matrix degrading gene variants: a prospective genetic association study. BMC Musculoskelet Disord. 2013;14:105 10.1186/1471-2474-14-105 23522322 PMC3610293

[22] Gendron C, Kashiwagi M, Lim NH, Enghild JJ, Thogersen IB, Hughes C, et al Proteolytic activities of human ADAMTS-5: comparative studies with ADAMTS-4. J Biol Chem. 2007;282(25):18294–18306. 10.1074/jbc.M701523200 17430884

[23] Liu S, Wu N, Liu J, Liu H, Su X, Liu Z, et al Association between ADAMTS-4 gene polymorphism and lumbar disc degeneration in Chinese Han population. J Orthop Res. 2016;34(5):860–864. 10.1002/jor.23081 26495885

[24] Wu N, Chen J, Liu H, Zhao L, Liu S, Liu J, et al The involvement of ADAMTS-5 genetic polymorphisms in predisposition and diffusion tensor imaging alterations of lumbar disc degeneration. J Orthop Res. 2014;32(5):686–694. 10.1002/jor.22582 24415654

[25] Mayer JE, Iatridis JC, Chan D, Qureshi SA, Gottesman O, Hecht AC. Genetic polymorphisms associated with intervertebral disc degeneration. The spine journal: official journal of the North American Spine Society. 2013;13(3):299–317. 23537453 10.1016/j.spinee.2013.01.041 PMC3655694

[26] Horvath G, Koroknai G, Acs B, Than P, Illes T. Prevalence of low back pain and lumbar spine degenerative disorders. Questionnaire survey and clinical-radiological analysis of a representative Hungarian population. Int Orthop. 2010;34(8):1245–1249. 10.1007/s00264-009-0920-0 19997731 PMC2989066

[27] Manek NJ, MacGregor AJ. Epidemiology of back disorders: prevalence, risk factors, and prognosis. Curr Opin Rheumatol. 2005;17(2):134–140. 15711224 10.1097/01.bor.0000154215.08986.06

[28] Walker BF, Williamson OD. Mechanical or inflammatory low back pain. What are the potential signs and symptoms? Man Ther. 2009;14(3):314–320. 10.1016/j.math.2008.04.003 18555728

[29] Kent P, Marks D, Pearson W, Keating J. Does clinician treatment choice improve the outcomes of manual therapy for nonspecific low back pain? A metaanalysis. J Manipulative Physiol Ther. 2005;28(5):312–322. 10.1016/j.jmpt.2005.04.009 15965405

[30] Omair A, Holden M, Lie BA, Reikeras O, Brox JI. Treatment outcome of chronic low back pain and radiographic lumbar disc degeneration are associated with inflammatory and matrix degrading gene variants: a prospective genetic association study. BMC Musculoskelet Disord. 2013;14:105 10.1186/1471-2474-14-105 23522322 PMC3610293

[31] Lane NE, Nevitt MC, Genant HK, Hochberg MC. Reliability of new indices of radiographic osteoarthritis of the hand and hip and lumbar disc degeneration. J Rheumatol. 1993;20(11):1911–1918. 8308778

[32] Arambepola C, Ekanayake R, Fernando D. Gender differentials of abdominal obesity among the adults in the district of Colombo, Sri Lanka. Prev Med. 2007;44(2):129–134. 10.1016/j.ypmed.2006.11.004 17178145

[33] Katulanda P, Jayawardena MA, Sheriff MH, Constantine GR, Matthews DR. Prevalence of overweight and obesity in Sri Lankan adults. Obes Rev. 2010;11(11):751–756. 10.1111/j.1467-789X.2010.00746.x 20406417

[34] Takatalo J, Karppinen J, Niinimaki J, Taimela S, Nayha S, Jarvelin MR, et al Prevalence of degenerative imaging findings in lumbar magnetic resonance imaging among young adults. Spine. 2009;34(16):1716–1721. 10.1097/BRS.0b013e3181ac5fec 19770614

[35] Pfirrmann CW, Metzdorf A, Zanetti M, Hodler J, Boos N. Magnetic resonance classification of lumbar intervertebral disc degeneration. Spine. 2001;26(17):1873–1878. 11568697 10.1097/00007632-200109010-00011

[36] Yu LP, Qian WW, Yin GY, Ren YX, Hu ZY. MRI assessment of lumbar intervertebral disc degeneration with lumbar degenerative disease using the Pfirrmann grading systems. PLoS One. 2012;7(12):e48074 10.1371/journal.pone.0048074 23284612 PMC3527450

[37] Gibbs RA, Belmont JW, Hardenbol P, Willis TD, Yu F, Yang H, et al The international HapMap project. Nature. 2003;426(6968):789–796. 10.1038/nature02168 14685227

[38] Qiagen QIAamp^®^ DNA Mini and Blood Mini Handbook. 2012. https://www.qiagen.com/lk/shop/sample-technologies/dna/dna-preparation/qiaamp-dna-blood-mini-kit/#resources.

[39] Promega. Measuring dsDNA Concentration Using the Quantus^™^ Fluorometer with the QuantiFluor^®^ dsDNA System 2014. http://worldwide.promega.com/resources/pubhub/applications-notes/measuring-dsdna-using-the-quantus-fluorometer-and-quantifluor-dsdna-dye/?activeTab=0.

[40] Gabriel S, Ziaugra L, Tabbaa D. SNP genotyping using the Sequenom MassARRAY iPLEX platform. Current protocols in human genetics / editorial board, Haines Jonathan L[et al]. 2009;Chapter 2:Unit 2.12. 10.1002/0471142905.hg0212s60 19170031

[41] Purcell S, Neale B, Todd-Brown K, Thomas L, Ferreira MA, Bender D, et al PLINK: a tool set for whole-genome association and population-based linkage analyses. Am J Hum Genet. 2007;81(3):559–575. 10.1086/519795 17701901 PMC1950838

[42] Choi Y, Sims GE, Murphy S, Miller JR, Chan AP. Predicting the functional effect of amino acid substitutions and indels. PloS one. 2012;7(10):e46688 10.1371/journal.pone.0046688 23056405 PMC3466303

[43] Kumar P, Henikoff S, Ng PC. Predicting the effects of coding non-synonymous variants on protein function using the SIFT algorithm. Nature protocols. 2009;4(7):1073–1081. 10.1038/nprot.2009.86 19561590

[44] Adzhubei IA, Schmidt S, Peshkin L, Ramensky VE, Gerasimova A, Bork P, et al A method and server for predicting damaging missense mutations. Nature methods. 2010;7(4):248–249. 10.1038/nmeth0410-248 20354512 PMC2855889

[45] Schwarz JM, Cooper DN, Schuelke M, Seelow D. MutationTaster2: mutation prediction for the deep-sequencing age. Nature methods. 2014;11(4):361–362. 10.1038/nmeth.2890 24681721

[46] Battie MC, Videman T, Parent E. Lumbar disc degeneration: epidemiology and genetic influences. Spine. 2004;29(23):2679–2690. 15564917 10.1097/01.brs.0000146457.83240.eb

[47] Adams MA, Lama P, Zehra U, Dolan P. Why do some intervertebral discs degenerate, when others (in the same spine) do not? Clin Anat. 2015;28(2):195–204. 10.1002/ca.22404 24753325

[48] Chan D, Song Y, Sham P, Cheung KM. Genetics of disc degeneration. Eur Spine J. 2006;15 Suppl 3:S317–325. 16819621 10.1007/s00586-006-0171-3 PMC2335375

[49] Sivan SS, Hayes AJ, Wachtel E, Caterson B, Merkher Y, Maroudas A, et al Biochemical composition and turnover of the extracellular matrix of the normal and degenerate intervertebral disc. Eur Spine J. 2014;23 Suppl 3:S344–353. 23591805 10.1007/s00586-013-2767-8

[50] Roughley P, Martens D, Rantakokko J, Alini M, Mwale F, Antoniou J. The involvement of aggrecan polymorphism in degeneration of human intervertebral disc and articular cartilage. European cells & materials. 2006;11:1–7; discussion 7. 16425147

[51] Eskola PJ, Kjaer P, Daavittila IM, Solovieva S, Okuloff A, Sorensen JS, et al Genetic risk factors of disc degeneration among 12-14-year-old Danish children: a population study. International journal of molecular epidemiology and genetics. 2010;1(2):158–165. 21537388 PMC3076758

[52] Sims AM, Timms AE, Bruges-Armas J, Burgos-Vargas R, Chou CT, Doan T, et al Prospective meta-analysis of interleukin 1 gene complex polymorphisms confirms associations with ankylosing spondylitis. Ann Rheum Dis. 2008;67(9):1305–1309. 10.1136/ard.2007.081364 18063673

[53] Johnsen AK, Plenge RM, Butty V, Campbell C, Dieguez-Gonzalez R, Gomez-Reino JJ, et al A broad analysis of IL1 polymorphism and rheumatoid arthritis. Arthritis Rheum. 2008;58(7):1947–1957. 10.1002/art.23592 18576312 PMC2533126

[54] Vo NV, Hartman RA, Yurube T, Jacobs LJ, Sowa GA, Kang JD. Expression and regulation of metalloproteinases and their inhibitors in intervertebral disc aging and degeneration. The spine journal: official journal of the North American Spine Society. 2013;13(3):331–341. 23369495 10.1016/j.spinee.2012.02.027 PMC3637842

[55] Nasto LA, Wang D, Robinson AR, Clauson CL, Ngo K, Dong Q, et al Genotoxic stress accelerates age-associated degenerative changes in intervertebral discs. Mechanisms of ageing and development. 2013;134(1–2):35–42. 10.1016/j.mad.2012.11.002 23262094 PMC3558562

[56] Furtwangler T, Chan SC, Bahrenberg G, Richards PJ, Gantenbein-Ritter B. Assessment of the matrix degenerative effects of MMP-3, ADAMTS-4, and HTRA1, injected into a bovine intervertebral disc organ culture model. Spine. 2013;38(22):E1377–1387. 10.1097/BRS.0b013e31829ffde8 23778376

[57] Rodriguez-Lopez J, Mustafa Z, Pombo-Suarez M, Malizos KN, Rego I, Blanco FJ, et al Genetic variation including nonsynonymous polymorphisms of a major aggrecanase, ADAMTS-5, in susceptibility to osteoarthritis. Arthritis Rheum. 2008;58(2):435–441. 10.1002/art.23201 18240210

[58] El Khoury L, Posthumus M, Collins M, Handley CJ, Cook J, Raleigh SM. Polymorphic variation within the ADAMTS2, ADAMTS14, ADAMTS5, ADAM12 and TIMP2 genes and the risk of Achilles tendon pathology: a genetic association study. Journal of science and medicine in sport / Sports Medicine Australia. 2013;16(6):493–498. 10.1016/j.jsams.2013.02.006 23491141

[59] Su CW, Huang YW, Chen MK, Su SC, Yang SF, Lin CW. Polymorphisms and Plasma Levels of Tissue Inhibitor of Metalloproteinase-3: Impact on Genetic Susceptibility and Clinical Outcome of Oral Cancer. Medicine (Baltimore). 2015;94(46):e2092. 26579821 10.1097/MD.0000000000002092 PMC4652830

[60] Gauderman WJ. Sample size requirements for association studies of gene-gene interaction. American journal of epidemiology. 2002;155(5):478–484. 11867360 10.1093/aje/155.5.478

