# Supplementary material for: Single Nucleotide Variants of Candidate Genes in Aggrecan Metabolic Pathway Are Associated with Lumbar Disc Degeneration and Modic Changes
Source: PLoS One. 2017 Jan 12;12(1):e0169835. doi: 10.1371/journal.pone.0169835 (PMC5231268; doi:10.1371/journal.pone.0169835)
Supplement: S1 Protocol — (DOCX) [file pone.0169835.s001.docx]

## Extraction of DNA from blood samples

DNA was extracted from peripheral blood using QIAamp DNA Mini Kit according to the blood and body fluid protocol of the user manual supplied with the kit [[1](#_ENREF_1)]. 20 µl of Qiagen protease was added in to a 1.5 ml micro-centrifuge tube. 200 µl of whole blood was added to the same tube and 200 µl of AL buffer was added and mixed by pulse vortexing for 15s to ensure efficient lysis. This solution was incubated in a water bath at 56 ºC for 10 minutes. Then the tube was briefly centrifuged to remove drops from the inside of the lid. 200 µl of ethanol was added to the sample and mixed again by pulse vortexing for 15 seconds followed by brief centrifugation. The mixture was carefully transferred into a QIAamp mini spin column (in a 2 ml collection tube) without wetting the rim. After closing the cap, the tube was centrifuged at 8000 rpm for 1 minute. Then the QIAamp mini spin column was placed in a clean 2 ml collection tube and the tube containing the filtrate was discarded. 500 µl of AW1 buffer was added without wetting the rim followed by centrifugation at 8000 rpm for 1 minute after closing the cap. Then the QIAamp mini spin column was placed in a clean 2 ml collection tube and the tube containing the filtrate was discarded. The spin column was carefully opened and 500 µl of AW2 buffer was added without wetting the rim. Then the mixture was centrifuged at 14,000 rpm for 3 minutes and the spin column was placed in a new 2 ml collection tube and the collection tube with the filtrate was discarded. The spin column with the collection tube was then centrifuged again at 14,000 rpm for 1 minute, following which the QIAamp mini spin column was placed in a clean 1.5 ml micro-centrifuge tube and the collection tube containing the filtrate was discarded. The QIAamp mini spin column was carefully opened and 200 µl of elusion buffer was added and incubated at room temperature for 1 minute. Then the mixture was centrifuged at 8000 rpm for 1 minute and the micro-centrifuge tube containing the eluted DNA was stored at -80°C.

## Quantification of extracted DNA

Extracted DNA was quantified using the Quantus fluorometer (Promega) with QuantiFluor®

Double stranded DNA (dsDNA) system according to the instructions in the user manual. The QuantiFluor® dsDNA System contains a fluorescent DNA binding dye which enables sensitive and specific quantification of small amounts of dsDNA [[2](#_ENREF_2)].

### Calibration of the Quantus fluorometer

The QuantiFluor® dsDNA working solution was prepared by diluting the dye at a 1:200 dilution in 1X TE buffer (eg. 10 µl of QuantiFluor® dsDNA dye to 1990 µl of 1X TE buffer) and mixing the solution. Blank solution for calibration was prepared by adding and mixing 100 μl of QuantiFluor® dsDNA dye working solution and 100 μl of 1X TE buffer to an empty 0.5 ml PCR tube. The standard solution was prepared by adding and mixing of 2 μl of provided DNA standard to 98 μl of 1X TE buffer. Then 100 μl was added from QuantiFluor® dsDNA dye working solution and mixed. The Quantus fluorometer was then calibrated by reading the blank and standard sample. Settings were saved to the instrument and re-calibration was done each time a quantification batch was processed [[2](#_ENREF_2)].

### Measuring an unknown sample

A volume of 1 μl of the unknown sample was diluted in a 99 μl of 1X TE buffer. 100 μl of QuantiFluor® dsDNA dye working solution was added to a 0.5 ml PCR tube and mixed for a total volume of 200 μl for quantification. The mixed solution was incubated at the room temperature for 5 minutes protected from light. The Quantus fluorometer was set to dsDNA protocol and volume of the unknown sample (1 μl) and the desired concentration units were selected. Then the concentration of unknown sample was measured [[2](#_ENREF_2)].

**References**

1. QIAGEN. QIAamp DNA Mini Blood Mini Handbook 2014. Available from: https://[www.qiagen.com/gb/shop/sample-technologies/dna/qiaamp-dna-mini-kit/#resources](http://www.qiagen.com/gb/shop/sample-technologies/dna/qiaamp-dna-mini-kit/#resources).

2. Promega Corporation. QuantiFluor® dsDNA System Technical Manual 2014 [cited 2014 15/03/]. Available from: https://worldwide.promega.com/resources/protocols/technical-manuals/101/quantifluor-dsdna-system-protocol/.
