# Supplementary material for: Single Nucleotide Variants of Candidate Genes in Aggrecan Metabolic Pathway Are Associated with Lumbar Disc Degeneration and Modic Changes
Source: PLoS One. 2017 Jan 12;12(1):e0169835. doi: 10.1371/journal.pone.0169835 (PMC5231268; doi:10.1371/journal.pone.0169835)
Supplement: S1 Table — (DOCX) [file pone.0169835.s002.docx]

**Table S1. Severity of MRI features tabulated according to genotype and the results of multiple linear regression.**

| **No.** | **Gene** | **SNV** | **Variable** | **A1** | **MAF** | **A2/A2** | **A1/A2** | **A1/A1** | **Regression  coefficient (β)** | **Adjusted  p value** |
| --- | --- | --- | --- | --- | --- | --- | --- | --- | --- | --- |
| 1 | *ADAMTS4* | rs34884997 |  | C | 0.12 |  |  |  |  |  |
|  |  |  | Genotypes |  |  | T/T | C/T | C/C |  |  |
|  |  |  | N |  |  | 80 | 23 | 2 |  |  |
|  |  |  | LDD mean (SD) |  |  | 12.34 (2.97) | 13.13 (2.56) | 14.00 (2.83) | 0.12 | 0.14 |
|  |  |  | Modic mean (SD) |  |  | 0.30 (1.05) | 1.04 (1.58) | 2.00 (0.00) | 0.31 | **0.00*** |
| 2 | *ADAMTS4* | rs41270041 |  | C | 0.18 |  |  |  |  |  |
|  |  |  | Genotypes |  |  | G/G | C/G | C/C |  |  |
|  |  |  | N |  |  | 69 | 35 | 1 |  |  |
|  |  |  | LDD mean (SD) |  |  | 12.72 (2.73) | 12.34 (3.10) | 7.00 (0.00) | -0.22 | **0.01*** |
|  |  |  | Modic mean (SD) |  |  | 0.51 (1.21) | 0.49 (1.29) | 0.00 (0.00) | -0.03 | 0.80 |
| 3 | *ADAMTS4* | rs4233367 |  | T | 0.18 |  |  |  |  |  |
|  |  |  | Genotypes |  |  | C/C | T/C | T/T |  |  |
|  |  |  | N |  |  | 72 | 29 | 4 |  |  |
|  |  |  | LDD mean (SD) |  |  | 12.68 (3.06) | 12.45 (2.40) | 10.75 (2.99) | -0.04 | 0.67 |
|  |  |  | Modic mean (SD) |  |  | 0.56 (1.34) | 0.34 (0.94) | 0.50 (1.00) | -0.07 | 0.50 |
| 4 | *ADAMTS4* | rs33941127 |  | T | 0.4 |  |  |  |  |  |
|  |  |  | Genotypes |  |  | C/C | T/C | T/T |  |  |
|  |  |  | N |  |  | 39 | 48 | 18 |  |  |
|  |  |  | LDD mean (SD) |  |  | 12.69 (2.88) | 12.21 (2.88) | 13.11 (2.97) | 0.10 | 0.26 |
|  |  |  | Modic mean (SD) |  |  | 0.75 (1.55) | 0.42 (1.09) | 0.17 (0.51) | -0.18 | 0.08 |
| 5 | *ADAMTS4* | rs34448954 |  | T | 0.13 |  |  |  |  |  |
|  |  |  | Genotypes |  |  | C/C | T/C | T/T |  |  |
|  |  |  | N |  |  | 81 | 20 | 3 |  |  |
|  |  |  | LDD mean (SD) |  |  | 12.42 (2.88) | 13.05 (3.02) | 12.67 (3.22) | 0.01 | 0.88 |
|  |  |  | Modic mean (SD) |  |  | 0.48 (1.11) | 0.25 (1.12) | 2.00 (3.46) | 0.08 | 0.42 |
| 6 | *IL1A* | rs2856836 |  | G | 0.27 |  |  |  |  |  |
|  |  |  | Genotypes |  |  | A/A | G/A | G/G |  |  |
|  |  |  | N |  |  | 58 | 38 | 9 |  |  |
|  |  |  | LDD mean (SD) |  |  | 13.09 (2.93) | 11.92 (2.72) | 11.67 (2.83) | -0.21 | **0.01*** |
|  |  |  | Modic mean (SD) |  |  | 0.69 (1.46) | 0.32 (0.87) | 0.00 (0.00) | -0.20 | **0.04*** |
| 7 | *IL1A* | rs1304037 |  | C | 0.28 |  |  |  |  |  |
|  |  |  | Genotypes |  |  | T/T | C/T | C/C |  |  |
|  |  |  | N |  |  | 58 | 37 | 10 |  |  |
|  |  |  | LDD mean (SD) |  |  | 13.09 (2.93) | 11.97 (2.73) | 11.5 (2.72) | -0.22 | **0.01*** |
|  |  |  | Modic mean (SD) |  |  | 0.69 (1.46) | 0.32 (0.88) | 0.00 (0.00) | -0.20 | **0.04*** |
| 8 | *IL1A* | rs17561 |  | A | 0.27 |  |  |  |  |  |
|  |  |  | Genotypes |  |  | C/C | A/C | A/A |  |  |
|  |  |  | N |  |  | 58 | 38 | 9 |  |  |
|  |  |  | LDD mean (SD) |  |  | 13.09 (2.93) | 11.92 (2.72) | 11.67 (2.83) | -0.21 | **0.01*** |
|  |  |  | Modic mean (SD) |  |  | 0.69 (1.46) | 0.32 (0.87) | 0.00 (0.00) | -0.20 | **0.04*** |
| 9 | *IL1A* | rs1800587 |  | A | 0.27 |  |  |  |  |  |
|  |  |  | Genotypes |  |  | G/G | A/G | A/A |  |  |
|  |  |  | N |  |  | 59 | 38 | 9 |  |  |
|  |  |  | LDD mean (SD) |  |  | 13.10 (2.91) | 12.11 (2.82) | 10.89 (2.03) | -0.23 | **0.00*** |
|  |  |  | Modic mean (SD) |  |  | 0.68 (1.46) | 0.32 (0.87) | 0.00 (0.00) | -0.20 | **0.04*** |
| 10 | *IL1B* | rs2853550 |  | A | 0.29 |  |  |  |  |  |
|  |  |  | Genotypes |  |  | G/G | A/G | A/A |  |  |
|  |  |  | N |  |  | 57 | 38 | 10 |  |  |
|  |  |  | LDD mean (SD) |  |  | 12.40 (2.70) | 12.55 (3.26) | 13.30 (2.54) | 0.05 | 0.55 |
|  |  |  | Modic mean (SD) |  |  | 0.67 (1.38) | 0.26 (0.92) | 0.40 (1.26) | -0.13 | 0.20 |
| 11 | *IL1B* | rs1143634 |  | A | 0.13 |  |  |  |  |  |
|  |  |  | Genotypes |  |  | G/G | A/G | A/A |  |  |
|  |  |  | N |  |  | 78 | 27 | 1 |  |  |
|  |  |  | LDD mean (SD) |  |  | 12.60 (2.84) | 12.41 (3.08) | 13.00 (0.00) | 0.01 | 0.92 |
|  |  |  | Modic mean (SD) |  |  | 0.54 (1.31) | 0.37 (0.97) | 0.00 (0.00) | -0.07 | 0.50 |
| 12 | *IL6* | rs1800796 |  | C | 0.46 |  |  |  |  |  |
|  |  |  | Genotypes |  |  | G/G | C/G | C/C |  |  |
|  |  |  | N |  |  | 30 | 56 | 20 |  |  |
|  |  |  | LDD mean (SD) |  |  | 12.13 (2.91) | 12.95 (2.86) | 12.10 (2.86) | 0.06 | 0.49 |
|  |  |  | Modic mean (SD) |  |  | 0.57 (1.48) | 0.59 (1.25) | 0.10 (0.45) | -0.14 | 0.18 |
| 13 | *IL6* | rs1800795 |  | C | 0.13 |  |  |  |  |  |
|  |  |  | Genotypes |  |  | G/G | C/G | C/C |  |  |
|  |  |  | N |  |  | 76 | 20 | 4 |  |  |
|  |  |  | LDD mean (SD) |  |  | 12.21 (2.78) | 13.85 (3.34) | 12.25 (1.89) | 0.10 | 0.24 |
|  |  |  | Modic mean (SD) |  |  | 0.28 (0.76) | 1.05 (1.76) | 0.00 (0.00) | 0.18 | 0.08 |
| 14 | *IL6* | rs2069849 |  |  | 0.05 |  |  |  |  |  |
|  |  |  | Genotypes | T |  | C/C | T/C | T/T |  |  |
|  |  |  | N |  |  | 94 | 11 | 0 |  |  |
|  |  |  | LDD mean (SD) |  |  | 12.63 (2.91) | 11.82 (2.68) | NA | -0.04 | 0.60 |
|  |  |  | Modic mean (SD) |  |  | 0.53 (1.28) | 0.18 (0.60) | NA | -0.08 | 0.42 |
| 15 | *MMP3* | rs520540 |  | A | 0.33 |  |  |  |  |  |
|  |  |  | Genotypes |  |  | G/G | A/G | A/A |  |  |
|  |  |  | N |  |  | 51 | 38 | 16 |  |  |
|  |  |  | LDD mean (SD) |  |  | 12.73 (3.29) | 12.84 (2.39) | 11.25 (2.35) | -0.07 | 0.39 |
|  |  |  | Modic mean (SD) |  |  | 0.61 (1.34) | 0.45 (1.16) | 0.25 (1.00) | -0.11 | 0.28 |
| 16 | *MMP3* | rs602128 |  | A | 0.33 |  |  |  |  |  |
|  |  |  | Genotypes |  |  | G/G | A/G | A/A |  |  |
|  |  |  | N |  |  | 51 | 38 | 16 |  |  |
|  |  |  | LDD mean (SD) |  |  | 12.73 (3.29) | 12.84 (2.39) | 11.25 (2.35) | -0.07 | 0.39 |
|  |  |  | Modic mean (SD) |  |  | 0.61 (1.34) | 0.45 (1.16) | 0.25 (1.00) | -0.11 | 0.28 |
| 17 | *MMP3* | rs679620 |  | T | 0.33 |  |  |  |  |  |
|  |  |  | Genotypes |  |  | C/C | T/C | T/T |  |  |
|  |  |  | N |  |  | 51 | 39 | 15 |  |  |
|  |  |  | LDD mean (SD) |  |  | 12.73 (3.29) | 12.77 (2.40) | 11.33 (2.41) | -0.07 | 0.41 |
|  |  |  | Modic mean (SD) |  |  | 0.61 (1.34) | 0.44 (1.14) | 0.27 (1.03) | -0.11 | 0.29 |
| 18 | *ACAN* | rs372041880 |  | C | 0.07 |  |  |  |  |  |
|  |  |  | Genotypes |  |  | A/A | C/A | C/C |  |  |
|  |  |  | N |  |  | 92 | 11 | 2 |  |  |
|  |  |  | LDD mean (SD) |  |  | 12.39 (2.91) | 13.36 (2.73) | 15.00 (0.00) | 0.11 | 0.19 |
|  |  |  | Modic mean (SD) |  |  | 0.52 (1.28) | 0.18 (0.60) | 1.00 (1.41) | -0.03 | 0.76 |
| 19 | *ACAN* | rs16942318 |  | A | 0.05 |  |  |  |  |  |
|  |  |  | Genotypes |  |  | C/C | A/C | A/A |  |  |
|  |  |  | N |  |  | 95 | 7 | 2 |  |  |
|  |  |  | LDD mean (SD) |  |  | 12.55 (2.98) | 12.71 (1.70) | 13.00 (2.83) | 0.04 | 0.67 |
|  |  |  | Modic mean (SD) |  |  | 0.48 (1.25) | 0.57 (0.98) | 1.00 (1.41) | 0.05 | 0.61 |
| 20 | *ACAN* | rs34949187 |  | A | 0.11 |  |  |  |  |  |
|  |  |  | Genotypes |  |  | G/G | A/G | A/A |  |  |
|  |  |  | N |  |  | 84 | 20 | 1 |  |  |
|  |  |  | LDD mean (SD) |  |  | 12.68 (2.90) | 11.95 (2.89) | 13.00 (0.00) | -0.05 | 0.58 |
|  |  |  | Modic mean (SD) |  |  | 0.55 (1.32) | 0.3 (0.73) | 0.00 (0.00) | -0.09 | 0.36 |
| 21 | *ACAN* | rs148070768 |  | G | 0.14 |  |  |  |  |  |
|  |  |  | Genotypes |  |  | A/A | G/A | G/G |  |  |
|  |  |  | N |  |  | 75 | 25 | 4 |  |  |
|  |  |  | LDD mean (SD) |  |  | 12.63 (2.70) | 11.88 (3.15) | 15.50 (3.70) | 0.06 | 0.45 |
|  |  |  | Modic mean (SD) |  |  | 0.49 (1.22) | 0.16 (0.55) | 2.75 (2.20) | 0.14 | 0.17 |
| 22 | *ACAN* | rs16942341 |  | T | 0.07 |  |  |  |  |  |
|  |  |  | Genotypes |  |  | C/C | T/C | T/T |  |  |
|  |  |  | N |  |  | 90 | 15 | 0 |  |  |
|  |  |  | LDD mean (SD) |  |  | 12.70 (2.90) | 11.60 (2.69) | NA | -0.14 | 0.09 |
|  |  |  | Modic mean (SD) |  |  | 0.42 (1.05) | 0.93 (1.98) | NA | 0.15 | 0.14 |
| 23 | *ACAN* | rs2272023 |  | C | 0.34 |  |  |  |  |  |
|  |  |  | Genotypes |  |  | A/A | C/A | C/C |  |  |
|  |  |  | N |  |  | 49 | 41 | 15 |  |  |
|  |  |  | LDD mean (SD) |  |  | 12.73 (3.13) | 12.15 (2.83) | 13.00 (2.14) | 0.00 | 1.00 |
|  |  |  | Modic mean (SD) |  |  | 0.65 (1.48) | 0.29 (0.84) | 0.53 (1.18) | -0.08 | 0.44 |
| 24 | *ACAN* | rs144501729 |  | A | 0.14 |  |  |  |  |  |
|  |  |  | Genotypes |  |  | C/C | A/C | A/A |  |  |
|  |  |  | N |  |  | 75 | 25 | 4 |  |  |
|  |  |  | LDD mean (SD) |  |  | 12.61 (2.70) | 11.88 (3.15) | 15.50 (3.70) | 0.07 | 0.41 |
|  |  |  | Modic mean (SD) |  |  | 0.47 (1.21) | 0.16 (0.55) | 2.75 (2.22) | 0.15 | 0.13 |
| 25 | *ACAN* | rs2351491 |  | T | 0.37 |  |  |  |  |  |
|  |  |  | Genotypes |  |  | C/C | T/C | T/T |  |  |
|  |  |  | N |  |  | 44 | 46 | 15 |  |  |
|  |  |  | LDD mean (SD) |  |  | 12.61 (2.86) | 12.43 (2.97) | 12.67 (2.87) | -0.02 | 0.81 |
|  |  |  | Modic mean (SD) |  |  | 0.64 (1.33) | 0.48 (1.28) | 0.13 (0.52) | -0.13 | 0.19 |
| 26 | *ACAN* | rs35430524 |  | A | 0.05 |  |  |  |  |  |
|  |  |  | Genotypes |  |  | C/C | A/C | A/A |  |  |
|  |  |  | N |  |  | 96 | 7 | 2 |  |  |
|  |  |  | LDD mean (SD) |  |  | 12.57 (2.92) | 11.57 (2.70) | 14.5 (0.71) | 0.04 | 0.61 |
|  |  |  | Modic mean (SD) |  |  | 0.51 (1.26) | 0.00 (0.00) | 1.50 (0.71) | 0.01 | 0.92 |
| 27 | *ACAN* | rs3743399 |  | G | 0.28 |  |  |  |  |  |
|  |  |  | Genotypes |  |  | A/A | G/A | G/G |  |  |
|  |  |  | N |  |  | 57 | 38 | 10 |  |  |
|  |  |  | LDD mean (SD) |  |  | 12.74 (2.96) | 12.13 (2.93) | 13.00 (2.31) | 0.03 | 0.77 |
|  |  |  | Modic mean (SD) |  |  | 0.56 (1.39) | 0.32 (0.87) | 0.80 (1.40) | 0.00 | 0.97 |
| 28 | *ACAN* | rs938609 |  | A | 0.37 |  |  |  |  |  |
|  |  |  | Genotypes |  |  | T/T | A/T | A/A |  |  |
|  |  |  | N |  |  | 44 | 45 | 15 |  |  |
|  |  |  | LDD mean (SD) |  |  | 12.61 (2.86) | 12.47 (3.00) | 12.67 (2.87) | -0.02 | 0.84 |
|  |  |  | Modic mean (SD) |  |  | 0.64 (1.33) | 0.49 (1.29) | 0.13 (0.52) | -0.13 | 0.20 |
| 29 | *ACAN* | rs2882676 |  | A | 0.47 |  |  |  |  |  |
|  |  |  | Genotypes |  |  | C/C | A/C | A/A |  |  |
|  |  |  | N |  |  | 32 | 47 | 26 |  |  |
|  |  |  | LDD mean (SD) |  |  | 12.88 (3.19) | 12.23 (2.94) | 12.69 (2.41) | -0.03 | 0.76 |
|  |  |  | Modic mean (SD) |  |  | 0.47 (1.22) | 0.47 (1.27) | 0.58 (1.21) | 0.03 | 0.75 |
| 30 | *ACAN* | rs28407189 |  | G | 0.07 |  |  |  |  |  |
|  |  |  | Genotypes |  |  | A/A | G/A | G/G |  |  |
|  |  |  | N |  |  | 90 | 15 | 0 |  |  |
|  |  |  | LDD mean (SD) |  |  | 12.70 (2.90) | 11.6 (2.69) | NA | -0.14 | 0.09 |
|  |  |  | Modic mean (SD) |  |  | 0.42 (1.05) | 0.93 (1.98) | NA | 0.15 | 0.14 |
| 31 | *ACAN* | rs79925540 |  | T | 0.16 |  |  |  |  |  |
|  |  |  | Genotypes |  |  | G/G | T/G | T/T |  |  |
|  |  |  | N |  |  | 74 | 27 | 4 |  |  |
|  |  |  | LDD mean (SD) |  |  | 12.59 (2.70) | 11.96 (3.09) | 15.5 (3.70) | 0.06 | 0.49 |
|  |  |  | Modic mean (SD) |  |  | 0.50 (1.23) | 0.15 (0.53) | 2.75 (2.22) | 0.12 | 0.21 |
| 32 | *ACAN* | rs4932439 |  | A | 0.28 |  |  |  |  |  |
|  |  |  | Genotypes |  |  | G/G | A/G | A/A |  |  |
|  |  |  | N |  |  | 57 | 38 | 10 |  |  |
|  |  |  | LDD mean (SD) |  |  | 12.74 (2.96) | 12.13 (2.93) | 13.00 (2.31) | 0.03 | 0.77 |
|  |  |  | Modic mean (SD) |  |  | 0.56 (1.39) | 0.32 (0.87) | 0.80 (1.40) | 0.00 | 0.97 |
| 33 | *ACAN* | rs3825994 |  | G | 0.44 |  |  |  |  |  |
|  |  |  | Genotypes |  |  | T/T | G/T | G/G |  |  |
|  |  |  | N |  |  | 34 | 51 | 20 |  |  |
|  |  |  | LDD mean (SD) |  |  | 12.59 (2.88) | 12.33 (2.96) | 13.00 (2.79) | 0.02 | 0.78 |
|  |  |  | Modic mean (SD) |  |  | 0.62 (1.35) | 0.51 (1.32) | 0.25 (0.64) | -0.10 | 0.31 |
| 34 | *ACAN* | rs76282091 |  | C | 0.16 |  |  |  |  |  |
|  |  |  | Genotypes |  |  | G/G | C/G | C/C |  |  |
|  |  |  | N |  |  | 74 | 27 | 4 |  |  |
|  |  |  | LDD mean (SD) |  |  | 12.59 (2.70) | 11.96 (3.09) | 15.50 (3.70) | 0.06 | 0.49 |
|  |  |  | Modic mean (SD) |  |  | 0.50 (1.23) | 0.15 (0.53) | 2.75 (2.22) | 0.12 | 0.21 |
| 35 | *ACAN* | rs1042630 |  | A | 0.41 |  |  |  |  |  |
|  |  |  | Genotypes |  |  | G/G | A/G | A/A |  |  |
|  |  |  | N |  |  | 40 | 45 | 20 |  |  |
|  |  |  | LDD mean (SD) |  |  | 12.98 (3.08) | 12.18 (2.97) | 12.50 (2.34) | -0.07 | 0.38 |
|  |  |  | Modic mean (SD) |  |  | 0.45 (1.13) | 0.58 (1.39) | 0.40 (1.05) | 0.00 | 1.00 |
| 36 | *ACAN* | rs1042631 |  | T | 0.35 |  |  |  |  |  |
|  |  |  | Genotypes |  |  | C/C | T/C | T/T |  |  |
|  |  |  | N |  |  | 45 | 47 | 14 |  |  |
|  |  |  | LDD mean (SD) |  |  | 12.98 (2.94) | 12.19 (2.96) | 12.43 (2.31) | -0.05 | 0.58 |
|  |  |  | Modic mean (SD) |  |  | 0.40 (1.07) | 0.55 (1.36) | 0.57 (1.22) | 0.06 | 0.54 |
| 37 | *ACAN* | rs698621 |  | G | 0.38 |  |  |  |  |  |
|  |  |  | Genotypes |  |  | T/T | G/T | G/G |  |  |
|  |  |  | N |  |  | 41 | 50 | 14 |  |  |
|  |  |  | LDD mean (SD) |  |  | 12.63 (2.93) | 12.36 (2.92) | 12.93 (2.79) | -0.01 | 0.90 |
|  |  |  | Modic mean (SD) |  |  | 0.68 (1.37) | 0.44 (1.23) | 0.14 (0.53) | -0.15 | 0.14 |
| 38 | *ACAN* | rs3817428 |  | G | 0.05 |  |  |  |  |  |
|  |  |  | Genotypes |  |  | C/C | G/C | G/G |  |  |
|  |  |  | N |  |  | 96 | 9 | 0 |  |  |
|  |  |  | LDD mean (SD) |  |  | 12.71 (2.90) | 10.78 (2.17) | NA | -0.17 | 0.05 |
|  |  |  | Modic mean (SD) |  |  | 0.54 (1.27) | 0.00 (0.00) | NA | -0.12 | 0.23 |
| 39 | *ACAN* | rs1126823 |  | G | 0.36 |  |  |  |  |  |
|  |  |  | Genotypes |  |  | A/A | G/A | G/G |  |  |
|  |  |  | N |  |  | 41 | 51 | 12 |  |  |
|  |  |  | LDD mean (SD) |  |  | 13.12 (2.54) | 12.00 (3.24) | 12.75 (2.18) | -0.09 | 0.31 |
|  |  |  | Modic mean (SD) |  |  | 0.46 (0.95) | 0.42 (1.20) | 1.00 (2.00) | 0.09 | 0.39 |
| 40 | *TIMP2* | rs2277698 |  | T | 0.24 |  |  |  |  |  |
|  |  |  | Genotypes |  |  | C/C | T/C | T/T |  |  |
|  |  |  | N |  |  | 60 | 40 | 5 |  |  |
|  |  |  | LDD mean (SD) |  |  | 12.63 (2.8) | 12.35 (3.01) | 13.00 (3.46) | -0.09 | 0.30 |
|  |  |  | Modic mean (SD) |  |  | 0.53 (1.31) | 0.50 (1.18) | 0.00 (0.00) | -0.06 | 0.54 |
| 41 | *ADAMTS5* | rs1444269 |  | G | 0.26 |  |  |  |  |  |
|  |  |  | Genotypes |  |  | A/A | G/A | G/G |  |  |
|  |  |  | N |  |  | 55 | 43 | 6 |  |  |
|  |  |  | LDD mean (SD) |  |  | 12.38 (3.05) | 12.91 (2.59) | 11.67 (3.67) | -0.04 | 0.66 |
|  |  |  | Modic mean (SD) |  |  | 0.51 (1.22) | 0.51 (1.32) | 0.33 (0.82) | -0.03 | 0.78 |
| 42 | *ADAMTS5* | rs2298657 |  | C | 0.05 |  |  |  |  |  |
|  |  |  | Genotypes |  |  | T/T | C/T | C/C |  |  |
|  |  |  | N |  |  | 94 | 9 | 0 |  |  |
|  |  |  | LDD mean (SD) |  |  | 12.6 (2.97) | 11.78 (2.11) | NA | -0.07 | 0.39 |
|  |  |  | Modic mean (SD) |  |  | 0.45 (1.17) | 1.11 (1.76) | NA | 0.15 | 0.10 |
| 43 | *ADAMTS5* | rs3746836 |  | A | 0.25 |  |  |  |  |  |
|  |  |  | Genotypes |  |  | G/G | A/G | A/A |  |  |
|  |  |  | N |  |  | 58 | 41 | 6 |  |  |
|  |  |  | LDD mean (SD) |  |  | 12.38 (2.98) | 12.90 (2.65) | 11.67 (3.67) | -0.03 | 0.76 |
|  |  |  | Modic mean (SD) |  |  | 0.48 (1.19) | 0.54 (1.34) | 0.33 (0.82) | -0.01 | 0.93 |
| 44 | *ADAMTS5* | rs229072 |  | T | 0.48 |  |  |  |  |  |
|  |  |  | Genotypes |  |  | A/A | T/A | T/T |  |  |
|  |  |  | N |  |  | 31 | 45 | 27 |  |  |
|  |  |  | LDD mean (SD) |  |  | 12.77 (3.04) | 12.84 (2.99) | 11.67 (2.50) | -0.05 | 0.59 |
|  |  |  | Modic mean (SD) |  |  | 0.42 (1.20) | 0.64 (1.40) | 0.37 (0.97) | -0.01 | 0.90 |
| 45 | *ADAMTS5* | rs229073 |  | G | 0.48 |  |  |  |  |  |
|  |  |  | Genotypes |  |  | A/A | G/A | G/G |  |  |
|  |  |  | N |  |  | 31 | 45 | 27 |  |  |
|  |  |  | LDD mean (SD) |  |  | 12.87 (3.06) | 12.84 (2.99) | 11.67 (2.50) | -0.06 | 0.51 |
|  |  |  | Modic mean (SD) |  |  | 0.35 (1.17) | 0.64 (1.40) | 0.37 (0.97) | 0.01 | 0.91 |
| 46 | *ADAMTS5* | rs11700721 |  | T | 0.12 |  |  |  |  |  |
|  |  |  | Genotypes |  |  | C/C | T/C | T/T |  |  |
|  |  |  | N |  |  | 81 | 23 | 1 |  |  |
|  |  |  | LDD mean (SD) |  |  | 12.33 (3.02) | 13.04 (2.12) | 18.00 (0.00) | 0.12 | 0.18 |
|  |  |  | Modic mean (SD) |  |  | 0.40 (1.06) | 0.87 (1.69) | 0.00 (0.00) | 0.13 | 0.20 |
| 47 | *ADAMTS5* | rs16979423 |  | G | 0.14 |  |  |  |  |  |
|  |  |  | Genotypes |  |  | T/T | G/T | G/G |  |  |
|  |  |  | N |  |  | 78 | 24 | 3 |  |  |
|  |  |  | LDD mean (SD) |  |  | 12.64 (2.88) | 12.62 (2.86) | 9.33 (2.08) | -0.13 | 0.12 |
|  |  |  | Modic mean (SD) |  |  | 0.59 (1.36) | 0.25 (0.68) | 0.00 (0.00) | -0.14 | 0.15 |
| 48 | *ADAMTS5* | rs9978597 |  | G | 0.05 |  |  |  |  |  |
|  |  |  | Genotypes |  |  | T/T | G/T | G/G |  |  |
|  |  |  | N |  |  | 94 | 9 | 0 |  |  |
|  |  |  | LDD mean (SD) |  |  | 12.46 (2.95) | 13.22 (2.33) | NA | 0.02 | 0.82 |
|  |  |  | Modic mean (SD) |  |  | 0.49 (1.15) | 0.00 (0.00) | NA | -0.12 | 0.23 |
| 49 | *ADAMTS5* | rs229078 |  | T | 0.22 |  |  |  |  |  |
|  |  |  | Genotypes |  |  | G/G | T/G | T/T |  |  |
|  |  |  | N |  |  | 64 | 35 | 5 |  |  |
|  |  |  | LDD mean (SD) |  |  | 12.95 (3.00) | 12.17 (2.63) | 10.00 (1.87) | -0.05 | 0.57 |
|  |  |  | Modic mean (SD) |  |  | 0.45 (1.26) | 0.54 (1.22) | 0.40 (0.89) | 0.03 | 0.80 |
| 50 | *ADAMTS5* | rs151065 |  | A | 0.19 |  |  |  |  |  |
|  |  |  | Genotypes |  |  | G/G | A/G | A/A |  |  |
|  |  |  | N |  |  | 66 | 36 | 3 |  |  |
|  |  |  | LDD mean (SD) |  |  | 11.98 (2.84) | 13.44 (2.73) | 14.00 (3.61) | 0.14 | 0.10 |
|  |  |  | Modic mean (SD) |  |  | 0.61 (1.39) | 0.33 (0.89) | 0.00 (0.00) | -0.13 | 0.19 |
| 51 | *ADAMTS5* | rs3746839 |  | G | 0.08 |  |  |  |  |  |
|  |  |  | Genotypes |  |  | A/A | G/A | G/G |  |  |
|  |  |  | N |  |  | 88 | 16 | 0 |  |  |
|  |  |  | LDD mean (SD) |  |  | 12.66 (3.01) | 12.00 (2.13) | NA | -0.15 | 0.08 |
|  |  |  | Modic mean (SD) |  |  | 0.52 (1.26) | 0.38 (1.09) | NA | -0.04 | 0.71 |
| 52 | *ADAMTS5* | rs226794 |  | A | 0.1 |  |  |  |  |  |
|  |  |  | Genotypes |  |  | G/G | A/G | A/A |  |  |
|  |  |  | N |  |  | 84 | 21 | 0 |  |  |
|  |  |  | LDD mean (SD) |  |  | 12.14 (2.92) | 14.14 (2.15) | NA | 0.20 | **0.02*** |
|  |  |  | Modic mean (SD) |  |  | 0.48 (1.26) | 0.57 (1.12) | NA | 0.03 | 0.75 |
| 53 | *ADAMTS5* | rs457947 |  | G | 0.07 |  |  |  |  |  |
|  |  |  | Genotypes |  |  | C/C | G/C | G/G |  |  |
|  |  |  | N |  |  | 92 | 12 | 1 |  |  |
|  |  |  | LDD mean (SD) |  |  | 12.61 (2.96) | 12.25 (2.30) | 10.00 (0.00) | 0.03 | 0.71 |
|  |  |  | Modic mean (SD) |  |  | 0.50 (1.24) | 0.50 (1.24) | 0.00 (0.00) | -0.02 | 0.84 |
| 54 | *ADAMTS5* | rs55933916 |  | G | 0.08 |  |  |  |  |  |
|  |  |  | Genotypes |  |  | C/C | G/C | G/G |  |  |
|  |  |  | N |  |  | 90 | 13 | 1 |  |  |
|  |  |  | LDD mean (SD) |  |  | 12.56 (2.92) | 12.31 (3.87) | 15.00 (0.00) | -0.02 | 0.82 |
|  |  |  | Modic mean (SD) |  |  | 0.40 (1.08) | 0.62 (1.26) | 6.00 (0.00) | 0.27 | **0.01*** |
| 55 | *TIMP3* | rs9862 |  | T | 0.5 |  |  |  |  |  |
|  |  |  | Genotypes |  |  | C/C | T/C | T/T |  |  |
|  |  |  | N |  |  | 23 | 58 | 23 |  |  |
|  |  |  | LDD mean (SD) |  |  | 13.26 (2.42) | 12.64 (3.06) | 11.61 (2.78) | -0.15 | 0.07 |
|  |  |  | Modic mean (SD) |  |  | 1.04 (1.58) | 0.40. (1.15) | 0.22 (0.85) | -0.23 | **0.02*** |
| 56 | *TIMP3* | rs11547635 |  | T | 0.07 |  |  |  |  |  |
|  |  |  | Genotypes |  |  | C/C | T/C | T/T |  |  |
|  |  |  | N |  |  | 93 | 11 | 1 |  |  |
|  |  |  | LDD mean (SD) |  |  | 12.38 (2.82) | 13.73 (3.35) | 15.00 (0.00) | 0.10 | 0.24 |
|  |  |  | Modic mean (SD) |  |  | 0.48 (1.19) | 0.64 (1.57) | 0.00 (0.00) | 0.02 | 0.86 |
| 57 | *TIMP3* | rs1427384 |  | C | 0.19 |  |  |  |  |  |
|  |  |  | Genotypes |  |  | T/T | C/T | C/C |  |  |
|  |  |  | N |  |  | 67 | 29 | 4 |  |  |
|  |  |  | LDD mean (SD) |  |  | 12.76 (2.78) | 11.9 (2.97) | 11.75 (3.30) | -0.10 | 0.25 |
|  |  |  | Modic mean (SD) |  |  | 0.36 (1.08) | 0.55 (1.06) | 0.00 (0.00) | 0.03 | 0.78 |
| 58 | *TIMP3* | rs2267184 |  | T | 0.16 |  |  |  |  |  |
|  |  |  | Genotypes |  |  | C/C | T/C | T/T |  |  |
|  |  |  | N |  |  | 76 | 26 | 3 |  |  |
|  |  |  | LDD mean (SD) |  |  | 12.82 (2.79) | 11.96 (3.08) | 10.67 (3.06) | -0.11 | 0.17 |
|  |  |  | Modic mean (SD) |  |  | 0.50 (1.30) | 0.54 (1.07) | 0.00 (0.00) | -0.02 | 0.81 |
| 59 | *TIMP3* | rs1065314 |  | C | 0.17 |  |  |  |  |  |
|  |  |  | Genotypes |  |  | T/T | C/T | C/C |  |  |
|  |  |  | N |  |  | 74 | 27 | 4 |  |  |
|  |  |  | LDD mean (SD) |  |  | 12.84 (2.78) | 11.85 (3.07) | 11.75 (3.30) | -0.11 | 0.20 |
|  |  |  | Modic mean (SD) |  |  | 0.49 (1.31) | 0.59 (1.08) | 0.00 (0.00) | -0.02 | 0.87 |
| 60 | *TIMP1* | rs5953060 |  | C | 0.43 |  |  |  |  |  |
|  |  |  | Genotypes |  |  | G/G | C/G | C/C |  |  |
|  |  |  | N |  |  | 43 | 34 | 28 |  |  |
|  |  |  | LDD mean (SD) |  |  | 12.09 (2.80) | 13.41 (3.01) | 12.18 (2.72) | 0.10 | 0.25 |
|  |  |  | Modic mean (SD) |  |  | 0.56 (1.40) | 0.62 (1.35) | 0.25 (0.65) | -0.04 | 0.71 |
| 61 | *TIMP1* | rs4898 |  | C | 0.43 |  |  |  |  |  |
|  |  |  | Genotypes |  |  | T/T | C/T | C/C |  |  |
|  |  |  | N |  |  | 43 | 34 | 28 |  |  |
|  |  |  | LDD mean (SD) |  |  | 12.09 (2.80) | 13.41 (3.01) | 12.18 (2.72) | 0.10 | 0.25 |
|  |  |  | Modic mean (SD) |  |  | 0.56 (1.4) | 0.62 (1.35) | 0.25 (0.65) | -0.04 | 0.71 |
| 62 | *TIMP1* | rs6609533 |  | G | 0.43 |  |  |  |  |  |
|  |  |  | Genotypes |  |  | A/A | G/A | G/G |  |  |
|  |  |  | N |  |  | 43 | 34 | 28 |  |  |
|  |  |  | LDD mean (SD) |  |  | 12.09 (2.80) | 13.41 (3.01) | 12.18 (2.72) | 0.10 | 0.25 |
|  |  |  | Modic mean (SD) |  |  | 0.56 (1.40) | 0.62 (1.35) | 0.25 (0.65) | -0.04 | 0.71 |

SNV – single nucleotide variation, LDD – lumbar disc degeneration, SD – standard deviation, A1 – minor allele, A2 – major allele, MAF – minor allele frequency, NA – not applicable, β – standardised regression coefficient, data were analysed by multiple linear regression on variant genotypes adjusting for age, gender and body mass index.

* - p value < 0.05
